# An insight into the temporal dynamics in the gut microbiome, metabolite signaling, immune response, and barrier function in suckling and weaned piglets under production conditions

**DOI:** 10.3389/fvets.2023.1184277

**Published:** 2023-08-31

**Authors:** Frederike Lerch, Fitra Yosi, Julia C. Vötterl, Simone Koger, Juliane Ehmig, Suchitra Sharma, Doris Verhovsek, Barbara U. Metzler-Zebeli

**Affiliations:** ^1^Unit of Nutritional Physiology, Institute of Physiology, Pathophysiology and Biophysics, Department of Biomedical Sciences, University of Veterinary Medicine Vienna, Vienna, Austria; ^2^Christian Doppler Laboratory for Innovative Gut Health Concepts of Livestock, Department for Farm Animals and Veterinary Public Health, Institute of Animal Nutrition and Functional Plant Compounds, University of Veterinary Medicine Vienna, Vienna, Austria; ^3^Department of Animal Science, Faculty of Agriculture, University of Sriwijaya, Palembang, South Sumatra, Indonesia; ^4^Institute of Animal Nutrition and Functional Plant Compounds, Department for Farm Animals and Veterinary Public Health, University of Veterinary Medicine Vienna, Vienna, Austria; ^5^Department for Farm Animals and Veterinary Public Health, University Clinic for Swine, University of Veterinary Medicine Vienna, Vienna, Austria

**Keywords:** bacteriome, host response, intestinal development, mycobiome, neonatal piglet, weaning, creep feeding

## Abstract

Little information is available on age- and creep-feeding-related microbial and immune development in neonatal piglets. Therefore, we explored age- and gut-site-specific alterations in the microbiome, metabolites, histo-morphology, and expression of genes for microbial signaling, as well as immune and barrier function in suckling and newly weaned piglets that were receiving sow milk only or were additionally offered creep feed from day of life (DoL) 10. The experiment was conducted in two replicate batches. Creep feed intake was estimated at the litter level. Piglets were weaned on day 28 of life. Gastric and cecal digesta and jejunal and cecal tissue were collected on DoL 7, 14, 21, 28, 31, and 35 for microbial and metabolite composition, histomorphology, and gene expression. In total, results for 10 piglets (*n* = 5/sex) per dietary group (sow milk only versus additional creep feed) were obtained for each DoL. The creep feed intake was low at the beginning and only increased in the fourth week of life. Piglets that were fed creep feed had less lactate and acetate in gastric digesta on DoL 28 compared to piglets fed sow milk only (*p* < 0.05). Age mainly influenced the gastric and cecal bacteriome and cecal mycobiome composition during the suckling phase, whereas the effect of creep feeding was small. Weaning largely altered the microbial communities. For instance, it reduced gastric *Lactobacillaceae* and cecal *Bacteroidaceae* abundances and lowered lactate and short-chain fatty acid concentrations on DoL 31 (*p* < 0.05). Jejunal and cecal expression of genes related to microbial and metabolite signaling, and innate immunity showed age-related patterns that were highest on DoL 7 and declined until DoL 35 (*p* < 0.05). Weaning impaired barrier function and enhanced antimicrobial secretion by lowering the expression of tight junction proteins and stimulating goblet cell recruitment in the jejunum and cecum (*p* < 0.05). Results indicated that age-dependent alterations, programmed genetically and by the continuously changing gut microbiome, had a strong impact on the expression of genes for gut barrier function, integrity, innate immunity, and SCFA signaling, whereas creep feeding had little influence on the microbial and host response dynamics at the investigated gut sites.

## Introduction

Neonatal microbial colonization is an important driver for porcine gut development; specifically for the build-up of barrier and immune functions (1). This development is interrupted by the removal of sow milk at weaning, leaving the piglet more susceptible to gut dysbiosis, inflammation, and a compromised barrier function (2). Sow milk contains many bioactive compounds that are crucial for the host gut development and shape the age-related development of the gut microbiota (3–5). These include fatty acids, bioactive peptides, biogenic amines, lactose, and oligosaccharides (3–5). Creep feeding is used for early gut training and acceptance of solid feed to ease the transition from sow milk to a plant-based diet, as well as to stimulate piglet’s feed intake post-weaning and change the gut microbiota toward microbes that utilize plant carbohydrates and proteins (6). Nevertheless, creep feed intake can be low and piglets can suffer from gut disorders and reduced nutrient absorption due to villus atrophy and crypt hyperplasia in the early post-weaning period (7).

Most knowledge about the successional changes in the gut microbial composition of newborn piglets has been gained by collecting feces (8–11), whereas information for other gut segments, such as the stomach and cecum, is less available. Moreover, knowledge about the effect of microbial development on microbial metabolite signaling and intestinal immunity from birth to the early post-weaning is still limited, especially with regard to the impact of creep feeding. Commensal fungi as part of the gut microbiome are essential for host immunity and health (12) but have been little investigated in the context of their postnatal development. The gut mucosa of the host senses microbial metabolites, such as short-chain (SCFA) and medium-chain fatty acids, via free fatty acid receptors (FFAR) along the gastrointestinal tract (13). Activation of these receptors generates numerous physiological effects, including anti-inflammatory effects (14). In addition, the host mucosa responds to microbial cell structures that activate the expression of pattern recognition receptors, such as toll-like receptors (TLR), which trigger inflammatory responses at the gut mucosa and are essentially involved in the build-up of immune tolerance during the suckling period (2, 15). Lately, bile acids (Bas) have been recognized as important regulators of inflammation and gut barrier function in piglets (16). Altered mucosal BA signaling due to undernutrition and low-fat content of the diet has been proposed to contribute to the inflammatory processes that occur at the gut mucosa of piglets in the first days postweaning (16), whereas little is known for the suckling period.

The objective of the present study was to explore age- and gut-site-specific alterations in the microbiome, microbial metabolites, histo-morphology, and expression of genes for microbial and BA signaling, as well as immune and barrier function in the gut of suckling and newly weaned piglets that were receiving sow milk only or were additionally offered creep feed from day of life (DoL) 10. We hypothesized that creep feeding would modulate the neonatal development of the microbiome and the host response related to microbial metabolite recognition and uptake as well as the mucosal innate immune response and barrier function. We assumed that creep feeding would increase microbial taxa capable of utilizing plant glycans and host tolerance toward a ‘plant-oriented’ microbiome, which should lead to a lower upregulation of inflammatory pathways and destruction of the epithelial integrity at the host mucosa after the dietary change at weaning.

## Materials and methods

### Animals and housing

The study was conducted under practical production conditions at the pig facility of the University of Veterinary Medicine Vienna (VetFarm). The sows were part of the regular sow herd, which included sows from parities 1–6. Management and feeding of sows and piglets corresponded to the routine protocol at the pig facility [see also (17)]. In two consecutive replicate batches, each lasting 35 days (from farrowing to one-week post-weaning), litters (Large White × Piétrain) from 20 Large White sows were used. Five days pre-farrowing, sows were moved to the farrowing barn where they were housed individually in BeFree pens (Schauer, Agrotonic, Prambachkirchen, Austria; 2.3 × 2.6 m). The pens were equipped with a bowl drinker, feeder, and hayrack for the sow and bowl drinkers and a nest with heated flooring for the piglets. The sows were not constrained during farrowing and farrowed within 48 h. Cross-fostering was applied only on DoL 1 to adjust the litter size to an average of 13 piglets. Mainly small birth weight piglets were cross-fostered to sows that were not included in the experiment. The other piglets remained with their respective mothers throughout lactation.

The health of the animals was checked visually each day. Piglets were weighed and identified with an ear tag after birth. Piglets received an iron injection on DoL 4 (2 mL Ferriphor 100 mg/mL, OGRIS Pharma Vetriebs-GmbH, Wels, Austria) and vaccination on DoL 17 (1 mL Ingelvac Circoflex plus 1 mL Ingelvac MycoFLEX, Boehringer Ingelheim RCV GmbH und Co KG, Vienna, Austria). Male piglets were castrated on DoL 11 after sedation (Stresnil 40 mg/mL, 0.025 mL/kg body weight, Elanco Tiergesundheit AG, Basel, Switzerland, and Narketan 100 mg/mL, 0.1 mL/kg body weight, Vetoquinol Österreich GmbH, Vienna, Austria). Male piglets recovered quickly and suckled normally in less than 6 h. Weaning occurred on DoL 28. Sows were first removed from the farrowing room before piglets were transferred to the weaner pig room. Weaned pigs were kept in groups of a maximum of 20 animals (pen size 3.3 m × 4.6 m), which meant that piglets from two to three litters of the same dietary group were housed together. Pens were equipped with a piglet nest, nipple and bowel drinkers, and one round feeder. Straw was provided as bedding material. Sows and piglets had always free access to water.

### Feeding and dietary groups

Throughout lactation, sows were fed a commercial cereal-soybean meal-based lactation diet according to the regular feeding protocol at the pig facility from 5 days before farrowing until weaning (Supplementary Table S1). The feed amount was gradually increased post-farrowing according to the regular feeding protocol. Thereafter, sows consumed the offered feed, which amounted to an average of 8.7 kg feed per sow and day. The litters were divided into two dietary groups, which were balanced for the parity of the sow. Per replicate batch, the piglets from five litters suckled only sow milk (sow milk group). The piglets from the other five litters per replicate batch additionally had free access to creep feed from day 10 of life (creep feed group). In total, for both replicate batches, there were 10 litters that only drank sow milk and 10 litters that had access to creep feed.

The creep feed was manually served and offered in piglet feed troughs at least twice daily, with sows having no access to the creep feed. The creep feed was a commercial milk replacer (Supplementary Table S1). Each litter received a minimum amount of 1,000 mL per day (500 mL at 08:00 and 500 mL at 15:00 h) in special stainless steel feeders, and more when piglets finished their portion. Leftover creep feed and spills were collected and recorded. Subsamples were taken for dry matter and nutrient analysis and the creep feed intake was estimated on a litter basis daily. The creep feed was prepared as described in a previous study (17). The dry powder of the milk replacer was mixed 1:5 (wt/vol; 200 g/L) with warm water (45°C) to achieve a thin liquid according to the manufacturer’s instructions. Afterward, the milk replacer cooled down and was fed at ambient temperature. On DoL 24 and 25, the creep feed was gradually mixed with the pre-starter diet (Supplementary Table S1) and fed as mash. From DoL 26 until weaning, the piglets from the creep feed group were fed 100% of the pre-starter diet in dry form. After weaning on DoL 28, the pre-starter diet was fed to 100% of the piglets in the creep feed group in dry form until the end of the experiment on DoL 35. Litters in the sow milk group did not receive the pre-starter diet before weaning and were offered the pre-starter diet in dry form from weaning (DoL 28) to DoL35. Piglets in both feeding groups had free access to the pre-starter diet postweaning. All diets were commercial complete feeds and met the current recommendations for nutrient requirements (18).

### Body weight measurement and gut sampling

Piglets were weighed immediately post-farrowing and on DoL 6, 13, 20, 27, 30, and 34. Piglets were not weighed on DoL 9 and 10 before starting with the creep feeding in order to keep piglets’ stress at lower levels, and in this way avoid piglets from refraining from starting to eat the creep feed due to a potential negative association with the weighing. This was also done considering the castration of male piglets on DoL 11. The difference in birth date was considered when calculating the average daily gain.

In each of the two replicate batches, gut samples were collected from 10 piglets each on DoL 7, 14, 21, 28, 31, and 35. In each replicate batch and at each of the six sampling days, five piglets of average body weight development per dietary group were selected. Birth weight and day and body weights on previous weighing days were also considered for the selection of the piglets. That is, one piglet per litter was selected on each sampling day. The selection of piglets on each sampling day was balanced for sex. In addition, it was taken care that the selection of males and females from each litter alternated. In total, there were observations from 10 piglets (5 male and 5 female piglets) per dietary group on each DoL.

**Figure 1:**
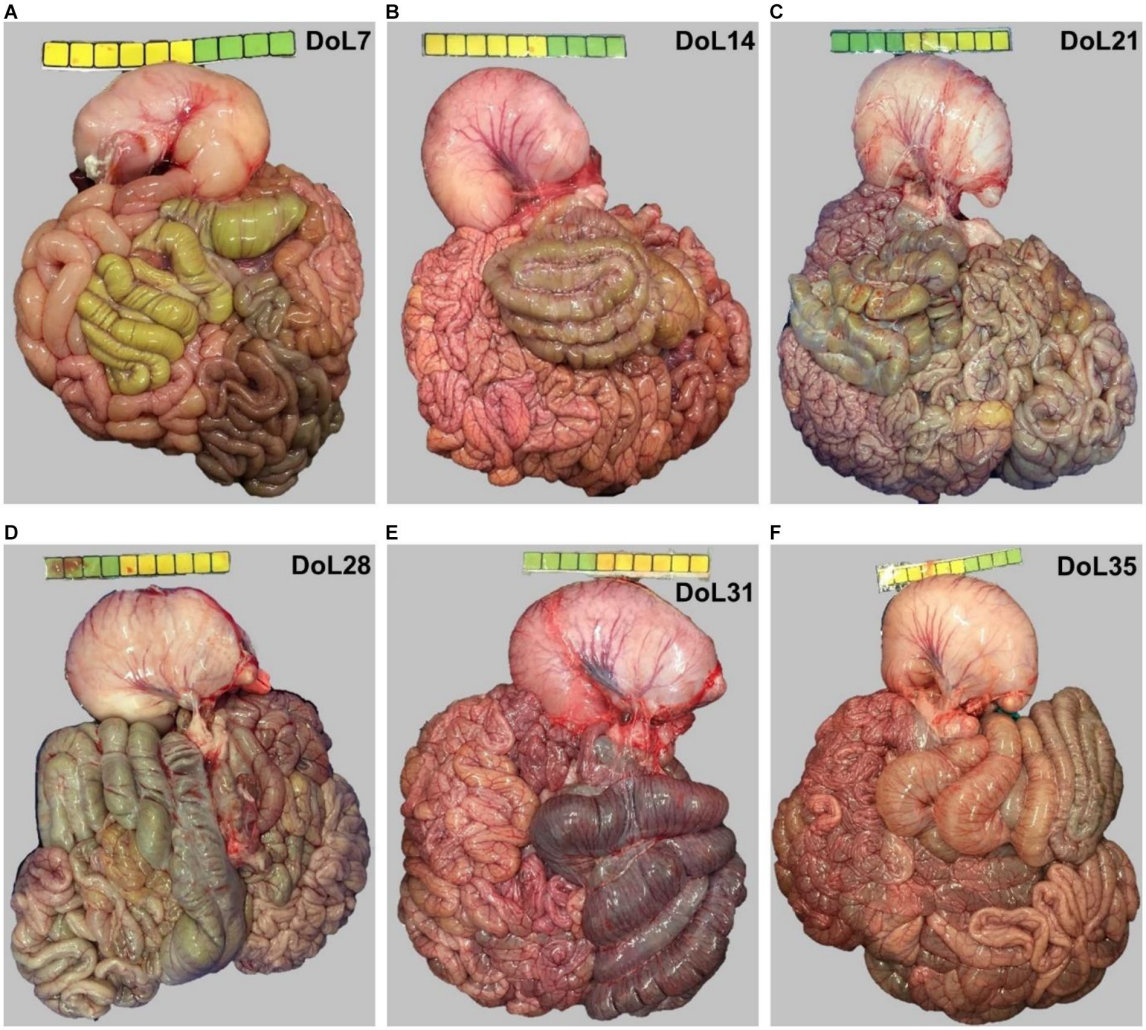
The gastrointestinal tract of piglets at day of life **(A)** 7, **(B)** 14, **(C)** 21, **(D)** 28, **(E)** 31, and **(F)** 35 with a scale (yellow-green) of 10 cm for comparison of intestinal length. DoL, day of life.

Before sampling, the piglet was weighed and anesthetized into the ear vein with azaperone (Stresnil 40 mg/mL, Elanco Tiergesundheit AG, Basel, Switzerland) and ketamine (Narketan 100 mg/mL, Vetoquinol Österreich GmbH, Vienna, Austria), and euthanized with an intracardiac application of embutramide (T61, Intervet GesmbH, Vienna, Austria) after complete sedation was ensured. The piglet was bled, the abdomen opened and the whole gut was removed aseptically. Pictures from the gut convolute were taken and the different parts were identified and clamped (Figure 1). The lengths of the stomach and cecum were measured as a proxy for intestinal development (19). Afterward, individual gut segments were clamped and separated. The mid-jejunum was selected as a representative gut segment for host digestive and absorptive functions. This segment was identified by dividing the total jejunum by two. The cecum was sampled as a major fermentative site in the gut of pigs. A 1-cm tube piece from the mid-jejunum and the tip of the cecal blind sack was taken for histomorphological measurements, fixated in neutral buffered 4%-formalin solution, and stored at 4°C. Digesta from the various gut sites were collected, homogenized, and snap-frozen in liquid nitrogen. To assess the gene expression, samples from the mid-jejunum (5-cm tube piece) and cecum (5 cm × 5 cm piece from the middle) were excised, washed in ice-cold phosphate-buffered saline, cut into small pieces, and snap-frozen. Samples were stored at −80°C.

### DNA extraction and determination of total microbial abundances

To assess gene copy numbers as a proxy for the total microbial load, total DNA was extracted from 250 mg gastric and cecal digesta using the DNeasy PowerSoil Kit (Qiagen, Hilden, Germany) with a few modifications to the manufacturer’s protocol. We did not obtain jejunal digesta from all piglets and therefore did not include this gut segment in the analysis. The modifications included an additional heating step for 10 min at 90°C and the homogenization of the samples using the SpeedMill Plus System (Analytik Jena GmbH, Jena, Germany). The DNA concentration in each eluate was quantified using the Qubit DNA HS Assay Kit on the Qubit 4 Fluorometer (Thermo Fisher Scientific Inc., Waltham, MA, United States).

Absolute quantification of total microbes in fecal samples was performed on a qTOWER real-time PCR system (Analytik Jena GmbH, Jena, Germany) using previously published primer sets (Supplementary Table S2). Each 20 μL reaction consisted of 5 ng DNA, 10 μL Eva Green master mix with low ROX (Biotium, Hayward, CA, United States), 400 nM each of forward and reverse primers, and 10 μL DEPC-treated water (Bioscience) in a 96-well plate. The amplification comprised an initial denaturation at 95°C for 5 min, followed by 40 cycles of 95°C for 15 s, primer annealing at 60°C for 30 s, and elongation at 72°C for 30 s, followed by the generation of melting curves with increments of 0.1°C/s between 55 and 95°C. Negative template controls were run in triplicates on each plate, whereas samples and the serial dilutions for the standard curves were run in duplicates. For the standard curves, 10-fold serial dilutions (10^7^–10^3^ molecules/μL) of the purified and quantified PCR products using pooled DNA from fecal samples of piglets from this study were prepared (20). The final copy numbers were calculated using the equation: (QM × C × DV)/ (S × V), where QM is the quantitative mean of the copy number, C is the DNA concentration of each sample, DV is the dilution volume of isolated DNA, S is the DNA amount (ng), and V is the weight of the sample (g) subjected to DNA extraction. Amplification efficiencies (*E* = 10^(−1/slope)^) and coefficient of determination (linearity) can be found in Supplementary Table S2.

### Sequencing and bioinformatics

The bacterial taxonomic composition was investigated in all gastric and cecal samples, whereas the taxonomic composition of fungi was only assessed at certain DoLs (*n* = 4), which were selected based on the results for the age-related development of the gene copy numbers of fungi and yeast from the quantitative PCR. For the determination of the bacterial and fungal taxonomy, the V3–V4 hypervariable regions of the bacterial 16S rRNA gene and the ITS2 region of fungi were amplified using a paired-end protocol (Microsynth, Balgach, Switzerland). Primers 341F-ill (5′-CCTACGGGNGGCWGCAG-3′) and 802R-ill (5′-GACTACHVGGGTATCTAATCC-3′) were used to amplify the V3-V4 region of the 16S rRNA gene and primers ITS3 (5′-GCATCGATGAAGAACGCAGC-3′), and ITS4 (5′-TCCTCCGCTTATTGATATGC-3′) were used for the ITS2 region. An amplicon of approximately 460 bp was generated for library preparation (Nextera XT DNA Sample Preparation Kit, Illumina, San Diego, CA, United States), and PCRs were performed using the KAPA HiFi HotStart PCR Kit (Roche, Baden, Switzerland).

Equimolar quantities of each library were pooled and sequenced on an Illumina MiSeq sequencing v2 platform using a paired-end protocol. Thereafter, reads were demultiplexed and adapter sequences were removed using cutadapt.^1^ The overlapping paired-end reads were stitched using USEARCH (drive5/com) by Microsynth.

Raw sequencing reads (Fastq files) for the 16S rRNA and fungal ITS amplicons were independently processed, aligned, and categorized using the Divisive Amplicon Denoising Algorithm 2 (DADA2; version 1.18.0) (21) in R studio (version 1.4.1106). Quality profiles of the forward and reverse reads were checked separately. The ‘file.path’ function was used to pre-filter sequences in order to remove reads with ambiguous bases. For the bacterial 16S rRNA amplicons, the first 10 nucleotides for each read were trimmed and the total length of forward and reverse reads were truncated to 220 nucleotides to account for the decrease in quality score of the further nucleotides using the ‘filterAndTrim’ function. For the fungal ITS amplicons, the first 10 nucleotides for each read were trimmed to account for the decrease in quality score of the following nucleotides, and a minimum length of 50 nucleotides was enforced to remove very low length sequences using the ‘filterAndTrim’ function. Moreover, reads exceeding the probabilistic estimated error of two nucleotides were removed in the same step (‘filterAndTrim’ function). After de-replication of the filtered data and estimation of error rates, amplicon sequence variants were inferred (21). Thereafter, the inferred forward and reverse sequences were merged using the function ‘mergepairs.’ This function merges the denoised pairs of forward and reverse reads, rejecting any pairs that do not sufficiently overlap or that contain too many mismatches in the overlap region. Chimera was removed using the ‘removeBimeraDenovo() function.’ Taxonomy was assigned using the function ‘assignTaxonomy,’ which implements the RDP Naive Bayesian Classifier algorithm and reference databases – SILVA 138 ribosomal RNA (rRNA) database for bacteria (22) and UNITE ITS database (version 9.0) for fungi (23) – with a dissimilarity threshold of 3%. The alpha diversity (Shannon, Simpson, Chao1) was determined using phyloseq (version 1.34.0).

### Determination of short-chain fatty acids

Gas chromatography (GC) was used to determine the SCFA in the gastric and cecal digesta as previously described (24). As mentioned above, we could not collect jejunal digesta from all piglets at all DoLs and did not analyze parameters in the few jejunal digesta samples. Acetate, propionate, butyrate, iso-butyrate, valerate, iso-valerate, and caproate were extracted from 1.0 g digesta with 25% ortho-phosphoric acid (4,36 mol/L) and 300 μL of 4-methylvaleric acid (23.83 μmol/mL) as internal standard (Sigma-Aldrich, St. Louis, MO, United States). Samples were briefly treated in an ultrasonic bath, mixed thoroughly, and centrifuged (20,000 × *g* 4°C for 20 min). Short-chain fatty acids were measured in the clear supernatant on the GC-2010 Plus Capillary GC (Shimadzu Corp., Kyoto, Japan) using a 30 m × 0.53 mm × 0.5 μm capillary column (Trace TR Wax, Thermo Fisher Scientific, Waltham, MA, United States) and helium as carrier gas. The gas chromatograph was equipped with an autosampler and injector (AOC-20s Auto Sampler; AOC-20i Auto-Injector, Shimadzu Corp., Kyoto, Japan) and a flame-ionization detector (FID-2010 Plus, Shimadzu Corp., Kyoto, Japan).

### Histo-morphological measurements

Preparation, imaging, and evaluation of histo-morphological parameters of the jejunal and cecal tissue were performed as previously described (25). Briefly, per gut site, piglet, and DoL, one discontinuous 3 to 4 μm-thick section was cut, deparaffinized, and stained with hematoxylin and eosin, and digital images were taken (Pannoramic Scan II slidescanner, 3DHISTECH Ltd., Budapest, Hungary) and analyzed (FIJI software) (26). Depending on the gut site, jejunal villus height and villus width were measured, whereas crypt depth, the circular and longitudinal muscle layer, count of goblet cells, and intraepithelial lymphocytes were obtained in the jejunum and cecum. In all, 15 measurements were taken from intact well-oriented, crypt-villus or crypt units for each feature. Villus height, villus width, crypt depth, and muscle layers were measured at 40 times magnification, and goblet cell and intraepithelial lymphocyte counts were measured at 100 times magnification.

### Gene expression analysis

To assess changes in mucosal gene expression, total RNA isolates from 24 mg frozen jejunal and cecal tissue were mechanically homogenized and analyzed using the RNeasy Mini Kit (RNeasy Mini Qiacube Kit, Qiagen, Hilden, Germany) (25). Each RNA isolate was treated with DNase I (Invitrogen TURBO DNA-free Kit, Thermo Fisher Scientific Inc.) to remove genomic DNA and quantified with the Qubit RNA HS Assay Kit on the Quibt 4 Fluorometer (Thermo Fisher Scientific Inc.). The quality of the RNA isolates was assessed with the Invitrogen Qubit RNA IQ Assay Kit (Thermo Fisher Scientific Inc.) on the Invitrogen Qubit 4 Fluorometer and only isolates with an RNA integrity number above eight were used for further gene expression analysis. Two μg of complementary DNA (cDNA) were synthesized from RNA with the AB High-Capacity cDNA Reverse Transcription Kit (Thermo Fisher Scientific Inc.) on the Mastercycler Nexus (Eppendorf SE, Hamburg, Germany) following the manufacturer’s protocol. The cDNA was analyzed for the expression of 19 target genes related to metabolite signaling, immune response, and barrier function and five reference genes as endogenous control [γ-actin (ACTG), hypoxanthine phosphoribosyl-transferase (HPRT), glyceraldehyde 3-phosphate-dehydrogenase (GAPDH), β2-microglobulin (B2M), and ornithine decarboxylase antizyme 1 (OAZ1)]. Primers used for the target gene and reference gene amplification were tested for their accuracy beforehand or newly designed with PrimerBLAST.^2^ Information about primers and their amplification efficiencies (*E* = 10^(−1/slope)^) are provided in Supplementary Table S3. A robot (epMotion 5075 TMX, Eppendorf SE, Hamburg, Deutschland) was used for qPCR pipetting and the innuMIX qPCR DS Green Standard (Analytik Jena, Jena, Germany) for amplification and quantification of cDNA in duplicates on the qTower384 (Analytik Jena, Jena, Germany). In each standard and sample reaction (10 μL), 7 μL master mix with innuMIX qPCR DS Green Standard, forward and reverse primers (150 nM each), and 3 μL cDNA template (25 ng) were included. After an initial denaturation step at 95°C for 2 min, 40 cycles of 95°C for 30 s, followed by primer annealing and elongation at 60°C for 30 s, were performed. Fluorescence was measured at all steps. Melting curves were generated to verify PCR amplification specificity. The geometric mean of the most stably expressed reference genes (ACTG, B2M, GAPDH, and OAZ1) was used to normalize raw gene expression and determine ΔCt values for relative gene expression levels. Relative gene expression was calculated with the 2^−ΔΔCt^ method (14). The ΔΔCt values were calculated using the sample with the highest expression (lowest ΔCt) of the respective target gene.

### Statistical and multivariate analyses

Normal distribution of the residuals of the data for body weight, gut microbiome, microbial metabolites, host mucosal gene expression, and histo-morphology was evaluated using the Shapiro–Wilk Test and UNIVARIATE procedure in SAS (Version 9.4; SAS Stat Inc., Cary, NC, United States). Afterward, data for body weight, gut microbiome, microbial metabolites, host mucosal gene expression, and histo-morphology (dependent variables) were subjected to ANOVA using the MIXED procedure in SAS and repeated measures were initially used to compare data between gut sites (for digesta-related parameters: stomach versus cecum; for tissue-related parameters: jejunum versus cecum). Thereafter, a random model was run separately per gut segment, including the fixed effects sex, replicate batch, DoL, feeding (sow milk only versus additional creep feed during the suckling phase), litter, and the respective two- and three-way interactions. The replicate batch and litter were considered as random effects and the piglet represented the experimental unit. A separate random model, which comprised the fixed effects replicate batch, sex, feeding, and the respective interactions as well as litter size at birth and birth date as covariates, was used to analyze the body weight development. The Kenward Roger method (dffm = kr) was used to approximate degrees of freedom. The probability of difference option in SAS was used to perform pairwise comparisons among least squares means. Data were expressed as least squares means ± standard error of the mean and differences, with *p* ≤ 0.05 and 0.05 < *p* ≤ 0.1 considered significant and as a trend, respectively. For the majority of parameters, differences between sexes were negligible and were removed from the final model. Descriptive statistics were calculated for creep feed intake during the suckling period using PROC MEANS in SAS. PROC CORR in SAS was used to calculate Pearson correlation coefficients between relative gene expression at the jejunal mucosa with SCFA concentrations in the gastric digesta, as well as cecal gene expression and cecal SCFA concentrations on DoL 7, 14, 21, 28, 31, and 35. Heat maps were generated using the packages ‘corrplot’ (27) and ‘ggplot2’ (28) in R Studio (version 2022.07.1).

Horizontal sparse partial least squares-discriminant analysis (sPLS-DA) using the ‘block.splsda’ function was applied to identify key features (most discriminant microbial taxa, i.e., bacteria and fungi, and expressed genes) and their relationships among datasets within the multigroup supervised DIABLO N-integration in the R package ‘mixOmics’(version 6.14.0) (29). The sPLS-DA was applied to integrate the datasets of relative abundances of bacterial and fungal genera in cecal digesta (datasets “Bacteria” and “Fungi”) and cecal expression levels of genes (dataset “Gene”) to identify and select key parameters from each dataset. To determine the main genera in digesta and mucosal expression levels of genes that allowed discrimination of dietary groups with the lowest possible error rate in the sPLS-DA, we tuned the number of retained variables. Data for bacterial genera and expressed mRNA were integrated for each DoL, whereas fungal data were only available for DoL 14, 28, 31, and 35 to be integrated with the two other datasets. We retained one-tenth of bacterial and fungal genera in cecal digesta (relative abundance >0.01%) and one-third of the most influential genes for components 1 and 2. The sPLS-DA results (*r* ≥ 0.4) were visualized as circos plots showing the strongest positive and negative Pearson’s correlations between most discriminant variables for each subset of data and component 1. Most discriminant variables for each subset of data and component 1 were visualized as loading plots.

## Results

### Creep feed intake, body weight, and gut development

Cross-fostering was used to adjust the number of piglets per litter on DoL 1. Subsequently, there were 12.8 and 12.4 ± 0.82 (standard deviation) piglets in the control and creep feeding groups, respectively, on DoL 2. The body weight was measured always 1 day before gut sampling to select the piglets with an average body weight within litters and feeding groups. Creep feed intake from DoL 10 varied among litters and the results for the descriptive statistics can be found in Supplementary Table S4. The estimated mean intake per piglet and day continuously increased from 10 g on DoL 10 to 79 g on DoL 28. Body weight was similar in both dietary groups, except for DoL 34 when the piglets from the creep-fed group weighed less than piglets only receiving sow milk (*p* = 0.005; Supplementary Table S5). The stomach and cecum lengths as proxies for gut development were affected by age but not by the feeding during the suckling phase. Both gut segments were greater on DoL 7, decreased to DoL 28, and increased again post-weaning (*p* < 0.001; Supplementary Table S2).

### Microbial composition in gastric and cecal digesta

**Figure 2:**
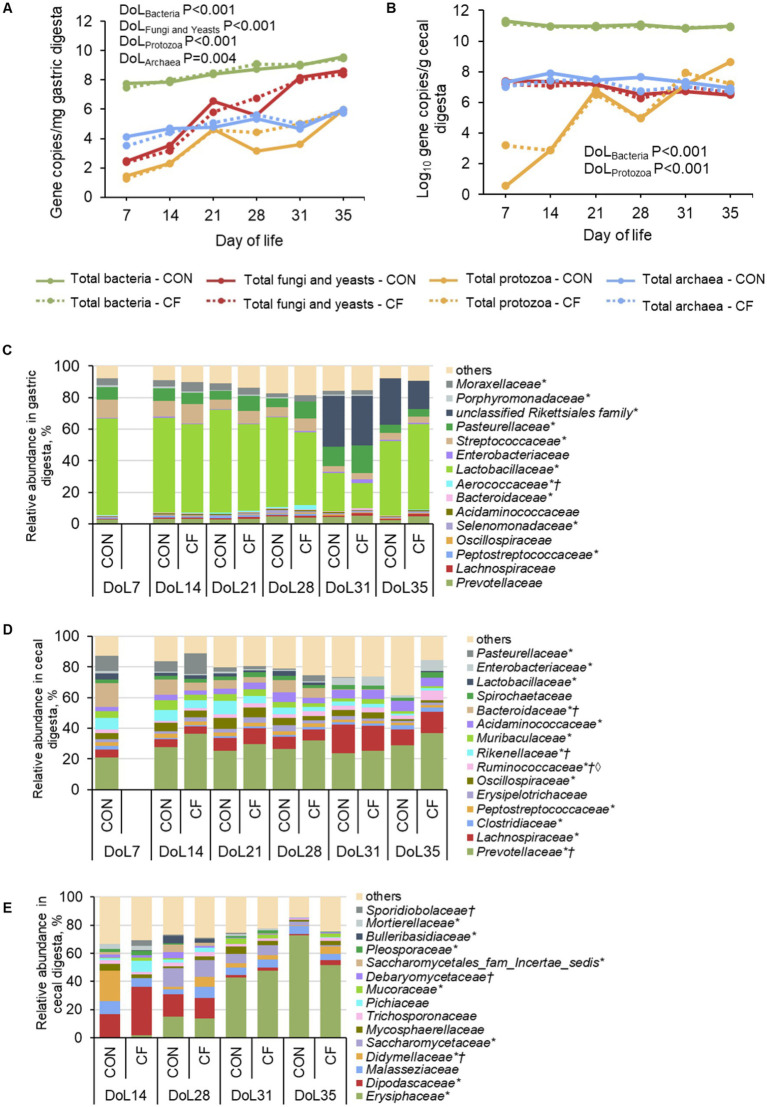
Age-related development of total microbial abundances and bacterial taxa in **(A,C)** gastric and **(B,D)** cecal digesta, respectively, as well as **(E)** fungal taxa in cecal digesta at family level in piglets receiving either sow milk only (CON) or additional creep feed from day 10 of life (CF). Results were obtained by quantitative PCR, 16S rRNA, and ITS sequencing, respectively. DoL, day of life. *Day of life effect *p* < 0.05; †Diet effect *p* < 0.05; ◊Day of life×diet effect *p* < 0.05. At each time point, 10 piglets (5 male piglets and 5 female piglets) per dietary group were sampled.

Quantitative PCR results revealed increasing gene copy numbers for bacteria, fungi, and yeasts, as well as protozoa and archaea in the gastric digesta from DoL 7–35 (*p* < 0.05; Figure 2A). In cecal digesta, bacterial numbers were highest on DoL 7 and protozoal numbers increased on DoL 21 and 31 compared to the other days, whereas archaea, fungi, and yeasts did not change (*p* < 0.001; Figure 2B). At both gut sites, the total abundances of bacteria, fungi, and yeasts, as well as protozoa and archaea were not affected by the creep feed. Bacterial species richness (Chao1) and diversity indices (Simpson and Shannon) increased during the suckling period and decreased post-weaning in the digesta of the stomach and cecum (*p* < 0.05), whereas they did not change for the fungal community in cecal digesta (Supplementary Figure S1). At both gut sites, diet did not affect alpha-diversity indices of the bacterial and fungal communities at the genus level. The 16S rRNA sequencing revealed that *Lactobacillaceae* predominated in the stomach during the suckling phase followed by *Streptococcaceae* and *Pasteurellaceae* (*p* < 0.05; Figure 2C). On DoL 31, gastric *Lactobacillaceae* and *Streptococcaceae* abundances declined, opening niches for the unclassified *Rikettsiales* family and *Pasteurellaceae*. This was reversed at DoL 35 when *Lactobacillaceae* increased again and the unclassified *Rikettsiales* family and *Pasteurellaceae* abundances decreased (*p* < 0.001). In cecal digesta, *Prevotellaceae* dominated during the suckling and post-weaning period, whereas *Bacteroidaceae* and *Pasteurellacae* were replaced by *Lachnospiraceae* and *Acidaminococcaceae* after weaning, respectively (*p* < 0.001; Figure 2D). Creep feed did not markedly affect the bacterial community in the stomach, except for increasing *Pasteurellaceae* on DoL 21 and 28 compared to the days before (*p* < 0.05). In the cecum, creep feeding caused a higher abundance of *Ruminococcaceae* at DoL 35 compared to the piglets that only received sow milk and the other sampling days (*p* = 0.02). The fungal composition in cecal digesta at DoL 14 was dominated by *Dipodascaceae*, which decreased with age and became low abundances post-weaning (*p* < 0.05; Figure 2E). *Erysiphaceae* was hardly present in cecal digesta on DoL 14 but increased on DoL 28 and became dominant after weaning (*p* < 0.001). *Saccharomycetaceae* appeared on DoL 28 in the cecal digesta, decreasing again after weaning (*p* < 0.05). Furthermore, *Didymellaceae* were lower in the cecum of creep-fed piglets than in piglets fed sow milk only on DoL 14 (*p* < 0.001). The sPLS-DA supported different discriminative bacteria and fungi in the cecal digesta of piglets that were fed only sow milk or were offered additional creep feed on the various DoLs during the suckling phase (Supplementary Figures S2, S3). Lower abundant bacterial taxa, in specific, discriminated at all ages, for instance, *Megasphaera* and *Dorea* for the creep-fed group on DoL 14 and 21, respectively. Postweaning, *Odoribacter* and *Escherichia/Shigella* were discriminative for the sow milk-only group on DoL 31 and 35, respectively. Regarding the fungal community in cecal digesta, *Aspergillus* discriminated for the creep-fed group on DoL 14, whereas *Didymella* and *Blumeria* discriminated on DoL 28 and 31 for the sow milk-only group, respectively. *Aspergillus* was again an influential taxon but for the sow milk-only group on DoL 35.

### pH, lactate, and short-chain fatty acid concentration in the gastric and cecal digesta

The pH of gastric and cecal digesta was stable during the suckling phase and declined when the piglet got older in both gut segments (*p* < 0.05). In the stomach, both lactate and acetate, as the major microbially produced FA, remained at a similar level during the suckling phase but largely decreased post-weaning (*p* < 0.001; Table 1). Creep feeding reduced gastric lactate and acetate concentrations by 49 and 37%, respectively, but only on DoL 28 (*p* < 0.05). In cecal digesta, concentrations of acetate, propionate, butyrate, and total SCFA increased until DoL 28, decreased on DoL 31 but increased again at DoL 35 (*p* < 0.001). Isobutyrate, valerate, isovalerate, and caproate concentrations increased until DoL 21 in the cecum, remained at a similar level on DoL 28, and decreased at DoL 31 (*p* < 0.001).

**Table 1:** Age-related development of total lactate and short-chain fatty acid (SCFA) concentrations in the gastric and cecal digesta of piglets receiving sow milk only or additional creep feed from day 10 of life.

Day of life (DoL)	DoL 7	DoL 14		DoL 21		DoL 28		DoL 31		DoL 35			*P*-value		
Feeding (Feed)	Sow milk	Sow milk	Creep feed	Sow milk	Creep feed	Sow milk	Creep feed	Sow milk	Creep feed	Sow milk	Creep feed	SEM	DoL	Feed	DoL×Feed
Stomach, μmol/g digesta															
pH	4.2	4.9	4.2	4.3	4.2	4.1	3.8	3.7	3.5	3.7	3.7	0.24	0.001	0.309	0.627
Total lactate	34.2	38.2	30.3	41.5	29.4	41.5	21.0	9.3	7.8	12.5	17.7	6.64	<0.001	0.460	0.071
Acetate	7.11	8.22	7.19	8.42	7.00	9.30	5.86	3.60	2.60	3.79	4.14	0.30	<0.001	0.009	0.418
Propionate	0	0.25	0.33	0.57	0.43	2.31	0.21	0	0	0	0	0.55	0.319	0.337	0.470
Total SCFA	7.11	8.47	7.52	8.99	7.42	12.86	6.09	3.60	2.60	3.79	4.14	1.40	0.0014	0.034	0.312
Cecum, μmol/g digesta															
pH	–	7.1	7.0	7.2	7.2	6.8	6.7	7.0	7.5	6.5	6.7	0.19	0.005	0.500	0.451
Total lactate	0	2.21	2.52	1.08	1.15	0.95	2.61	3.12	1.51	3.19	6.32	1.40	<0.001	0.450	0.553
Acetate	25.93	43.9	40.1	70.3	67.6	97.9	99.6	49.7	45.7	62.0	66.0	7.31	<0.001	0.982	0.983
Propionate	9.79	13.60	13.13	22.9	25.7	32.4	33.0	19.0	16.1	26.7	24.2	2.82	<0.001	0.661	0.906
Butyrate	3.17	5.24	4.78	9.66	9.76	15.07	16.1	5.88	5.25	10.47	8.28	1.53	<0.001	0.635	0.938
Isobutyrate	1.06	1.66	1.62	3.10	3.65	3.91	3.03	0.45	0.49	0.198	0.304	0.37	<0.001	0.765	0.477
Valerate	1.33	2.11	2.02	3.92	4.35	4.63	4.18	0.91	0.90	1.72	1.11	0.47	<0.001	0.570	0.891
Isovalerate	1.39	1.97	1.99	3.56	3.98	3.96	2.99	0.52	0.56	0.34	0.33	0.345	<0.001	0.476	0.400
Caproate	0	0.044	0.058	0.13	0.18	0.31	0.10	0.070	0.064	0.017	0.032	0.04	<0.001	0.370	0.008
Total SCFA	42.6	68.5	63.7	113.6	115.2	158.1	159.0	76.6	69.0	101.4	100.3	11.61	<0.001	0.811	0.998

Values are presented as least squares means ± standard error of the mean (SEM). Mainly acetate, propionate, and lactate were detected in gastric digesta. The amount of digesta on DoL 7 was not sufficient to allow measuring pH. At each time point, 10 piglets (5 male piglets and 5 female piglets) per dietary group were sampled.

### Jejunal and cecal histo-morphology

Histomorphological measures were performed using jejunal and cecal tissue samples, revealing gut site-specific characteristics of mucosal structures (Table 2). Time-point associated effects showed a gradual reduction of jejunal villus height and surface with age, lower apical villus width on DoL 21 and 35, and greater basal villus width on DoL 21, which decreased at DoL 35 compared to the DoL before (*p* < 0.05). Compared to the other DoLs during suckling, jejunal crypt depth increased at DoL 28 compared to the days before and was constant post-weaning (*p* < 0.001). Circular and longitudinal muscle thickness in the jejunum were enhanced at DoL 28 compared to the other days, the latter thinning again at DoL 35 (*p* < 0.001). The number of goblet cells in the jejunum increased at DoL 28 and decreased again at DoL 31, whereas the number of intraepithelial lymphocytes was higher at DoL 31 and lowered at DoL 35 compared to pre-weaning (*p* < 0.001). Cecal crypt depth gradually increased with age and circular muscle thickness at DoL 28 compared to the days before, whereas it decreased again post-weaning (*p* < 0.001). The numbers of goblet cells and intraepithelial lymphocytes in the cecum were highest on DoL 31 and lowered again on DoL 35 (*p* < 0.001). Creep feeding did not affect jejunal and cecal morphology.

**Table 2:** Age-related development of histomorphological measures in the jejunum and cecum of piglets receiving only sow milk or additional creep feed from day 10 of life.

Day of life (DoL)	7	14		21		28		31		35		*P*-value			
Feeding (Feed)	Sow milk	Sow milk	Creep feed	Sow milk	Creep feed	Sow milk	Creep feed	Sow milk	Creep feed	Sow milk	Creep feed	SEM	DoL	Feed	DoL×Feed
Jejunum															
Villus height, μm	1169	1067	906	895	705	484	536	353	279	360	388	83.4	<0.001	0.990	0.252
Apical villus width, μm	101	99	97	82	83	96	106	106	115	85	100	6.9	<0.001	0.331	0.322
Basal villus width, μm	119	114	104	123	130	116	130	131	136	111	128	9.2	0.013	0.195	0.397
Villus surface, mm^2^	1	0.73	0.60	0.59	0.48	0.32	0.40	0.26	0.22	0.23	0.28	0.075	<0.001	0.867	0.279
Villus height to crypt depth ratio	8	6.7	5.9	6.7	5.1	2.2	2.2	1.4	40.2	1.8	1.5	16.11	0.594	0.437	0.425
Crypt depth, μm	152	162	153	145	144	236	247	264	249	225	262	21.1	<0.001	0.706	0.556
Circular muscle, μm	53	77	73	76	73	144	124	134	125	121	142	19.3	<0.001	0.854	0.793
Longitudinal muscle, μm	40	42	39	44	56	105	90	79	67	70	76	13.3	<0.001	0.965	0.675
Number of goblet cells	7	6	5	3	4	7	9	3	3	3	4	0.8	<0.001	0.399	0.217
Number of intraepithelial lymphocytes	1	2	2	1	1	3	3	11	9	6	7	1.9	<0.001	0.986	0.906
Cecum															
Crypt depth, μm	269	300	310	324	306	366	381	397	367	436	424	20.8	<0.001	0.901	0.579
Circular muscle, μm	191	234	237	145	103	272	294	274	190	157	110	40.8	<0.001	0.722	0.471
Longitudinal muscle, μm	110	142	145	169	100	195	186	192	121	109	104	50.6	0.228	0.932	0.806
Number of goblet cells	21	22	23	20	20	22	24	35	32	19	23	2.8	<0.001	0.088	0.473
Number of intraepithelial lymphocytes	1	2	2	0	0	2	2	3	2	0	0	0.3	<0.001	0.617	0.357

Creep feeding did not affect intestinal structures in the jejunum and cecum. Values are presented as least squares means ± standard error of the mean. VH:CD ratio, villus height to crypt depth ratio; IEL, intraepithelial lymphocytes. Day of life effect *P* < 0.05. At each time point, 10 piglets (5 male piglets and 5 female piglets) per dietary group were sampled.

### Jejunal and cecal gene expression

Jejunal expression of *FFAR1* and *FFAR3* decreased on DoL 28 compared to the time points before (*p* < 0.001; Table 3). Expression of *FFAR2* and *FFAR4* in the jejunum declined gradually from DoL 7 to the other time points, with *FFAR4* expression increasing again post-weaning (*p* < 0.001). Jejunal *MCT1* expression was lowered from DoL 7–28, whereas expression of *SMCT1* and *HCAR1* decreased at DoL 28 and *FXR* at DoL 14; the latter being enhanced again at DoL 35 compared to the other time points (*p* < 0.05). Expression of *TLR1* and *TLR2* decreased at DoL 28 and DoL 14 in the jejunum, respectively, compared to the days before, whereas *TLR4* and *TLR9* expression gradually declined from DoL 14 to 28 (*p* < 0.05). Expression of *IAP* decreased from DoL 7–28 (*p* < 0.05) at the jejunal mucosa. Expression of *CLDN1* and *CLDN4* was not affected in the jejunum, while *OCLN* and *ZO1* expression decreased from DoL 14–28, with *OCLN* increasing again at DoL 35 (*p* < 0.001). In the cecum, *FFAR2* and *MCT1* were consistently expressed, whereas expression of *FFAR1* decreased at DoL 28 and that of *FFAR3* at DoL 14 and 31 compared to the DoLs before (*p* < 0.05; Table 4). Cecal expression of *FFAR4* also decreased at DoL 28 compared to the DoLs before, whereas it increased at DoL 31 and declined again on DoL 35 (*p* < 0.05). Cecal *HCAR1* expression increased at DoL 14 and decreased at DoL 28 (*p* < 0.001). *SMCT1* and *FXR* were highest expressed at DoL 7 compared to the other DoLs (*p* < 0.001). *TLR9* was stably expressed at the cecal mucosa across time points ( Table 4). Cecal *TLR1* expression was reduced at DoL 35 and *TLR2* at DoL 21, whereas *TLR4* expression was upregulated at DoL 21 and decreased at DoL 35 compared to the DoLs before (*p* < 0.01). Expression of tight junction proteins *CLDN1*, *CLDN4*, and *ZO1* in the cecum was lower at DoL 31, DoL 21, and DoL 28, respectively, compared to the other time points, and *OCLN* expression declined at DoL 14, 28, and 35 (*p* < 0.001). Cecal expression of *IAP* peaked at DoL 7 compared to the other days (*p* < 0.001). The expression of *MUC4* increased at DoL 31 and decreased again at DoL 35 (*p* < 0.001), whereas *MUC2* expression did not differ at the cecal mucosa. The sPLS-DA identified *FXR* and *IAP* as the most influentially expressed genes characterizing the sow milk-only group on DoL 14, whereas the expression of *SMCT1* and *ZO1* discriminated for the creep feeding group on DoL 21 and 28, respectively (Supplementary Figure S2). Postweaning, *TLR9* and *TLR2* expression discriminated for the sow milk-only group on DoL 31 and 35, respectively. Relationships between the most discriminative bacterial and fungal taxa and the expression levels of genes can be found in Supplementary Figure S3.

**Table 3:** Age-related development of the relative expression of genes for short-chain fatty acid receptors and transporters, bile acid receptors, pattern recognition receptors, antimicrobial secretions, and barrier function at the jejunal mucosa of piglets receiving only sow milk or additional creep feed from day 10 of life.

Day of life (DoL)	DoL 7	DoL 14		DoL 21		DoL 28		DoL 31		DoL 35		*P*-value			
Feeding (Feed)	Sow milk	Sow milk	Creep feed	Sow milk	Creep feed	Sow milk	Creep feed	Sow milk	Creep feed	Sow milk	Creep feed	SEM	DoL	Feed	DoL×Feed
Short-chain fatty acid receptors and transporters															
*FFAR1*	0.31	0.17	0.32	0.18	0.23	0.06	0.07	0.07	0.07	0.06	0.05	0.055	<0.001	0.217	0.215
*FFAR2*	0.44	0.35	0.44	0.28	0.30	0.23	0.17	0.13	0.16	0.13	0.11	0.049	<0.001	0.659	0.358
*FFAR3*	0.51	0.41	0.44	0.33	0.38	0.16	0.11	0.09	0.08	0.08	0.06	0.064	<0.001	0.971	0.904
*FFAR4*	0.08	0.06	0.07	0.05	0.06	0.02	0.02	0.02	0.03	0.04	0.03	0.010	<0.001	0.981	0.738
*HCAR1*	0.08	0.10	0.09	0.07	0.09	0.03	0.03	0.05	0.05	0.03	0.03	0.018	<0.001	0.606	0.836
*MCT1*	0.05	0.03	0.03	0.02	0.02	0.01	0.02	0.02	0.02	0.02	0.02	0.004	<0.001	0.736	0.470
*SMCT*	0.33	0.52	0.44	0.42	0.46	0.33	0.37	0.37	0.34	0.44	0.37	0.064	0.004	0.341	0.487
Bile acid receptor															
*FXR*	0.29	0.16	0.15	0.17	0.17	0.11	0.14	0.19	0.22	0.21	0.24	0.060	0.004	0.637	0.992
Pattern recognition receptors															
*TLR1*	0.14	0.12	0.13	0.11	0.12	0.06	0.07	0.07	0.08	0.07	0.07	0.023	<0.001	0.855	0.617
*TLR2*	0.09	0.06	0.03	0.02	0.02	0.03	0.02	0.02	0.04	0.02	0.01	0.021	<0.001	0.521	0.858
*TLR4*	0.15	0.16	0.15	0.11	0.12	0.11	0.09	0.09	0.11	0.10	0.11	0.023	0.004	0.797	0.480
*TLR9*	0.31	0.31	0.37	0.23	0.25	0.25	0.26	0.17	0.29	0.21	0.17	0.055	0.003	0.505	0.194
Antimicrobial secretion															
*IAP*	0.41	0.28	0.25	0.18	0.17	0.10	0.11	0.06	0.07	0.09	0.10	0.058	<0.001	0.887	0.945
*MUC2*	0.15	0.17	0.15	0.13	0.14	0.11	0.12	0.14	0.13	0.16	0.16	0.033	0.307	0.919	0.976
*MUC4*	0.002	0.002	0.002	0.001	0.002	0.001	0.001	0.001	0.001	0.001	0.126	0.0461	0.488	0.274	0.474
Tight junction proteins															
*CLDN1*	0.02	0.02	0.02	0.03	0.04	0.04	0.02	0.03	0.04	0.02	0.02	0.013	0.427	0.501	0.211
*CLDN4*	0.35	0.44	0.35	0.37	0.33	0.27	0.27	0.30	0.24	0.31	0.28	0.078	0.150	0.380	0.970
*OCLN*	0.30	0.45	0.35	0.29	0.34	0.20	0.21	0.25	0.18	0.30	0.38	0.063	<0.001	0.695	0.147
*ZO1*	0.40	0.38	0.32	0.27	0.26	0.20	0.18	0.17	0.21	0.20	0.18	0.058	<0.001	0.539	0.925

Values are presented as least squares means ± SEM. *TLR1*, toll-like receptor 1; *TLR2*, toll-like receptor 2; *TLR4*, toll-like receptor 4; *TLR9*, toll-like receptor 9; *IAP*, intestinal alkaline phosphatase; *MUC2*, mucin 2; *MUC4*, mucin 4; *CLDN1*, claudin 1; *CLDN4*, claudin 4; *OCLN*, occludin; *ZO1*, zonula occludens-1. Day of life effect *P* < 0.05. At each time point, 10 piglets (5 male piglets and 5 female piglets) per dietary group were sampled.

**Table 4:** Age-related development of the relative expression of genes for short-chain fatty acid receptors and transporters, bile acid receptors, pattern recognition receptors, antimicrobial secretion, and barrier function at the cecal mucosa of piglets receiving only sow milk or additional creep feed from day 10 of life.

Day of life (DoL)	7	14		21		28		31		35		*P*-value			
Feeding (Feed)	Sow milk	Sow milk	Creep feed	Sow milk	Creep feed	Sow milk	Creep feed	Sow milk	Creep feed	Sow milk	Creep feed	SEM	DoL	Feed	DoL×Feed
Short-chain fatty acid receptors and transporters															
*FFAR1*	0.05	0.05	0.05	0.05	0.06	0.04	0.03	0.03	0.02	0.01	0.03	0.013	0.002	0.909	0.336
*FFAR2*	0.35	0.31	0.33	0.28	0.32	0.25	0.24	0.24	0.29	0.34	0.30	0.082	0.481	0.782	0.604
*FFAR3*	0.14	0.09	0.09	0.10	0.12	0.08	0.08	0.04	0.05	0.03	0.04	0.014	<0.001	0.934	0.125
*FFAR4*	0.32	0.44	0.36	0.38	0.42	0.28	0.29	0.38	0.44	0.26	0.35	0.085	0.038	0.971	0.325
*HCAR1*	0.25	0.33	0.38	0.37	0.39	0.26	0.20	0.16	0.16	0.18	0.17	0.060	<0.001	0.861	0.800
*MCT1*	0.22	0.24	0.22	0.21	0.25	0.20	0.23	0.28	0.19	0.20	0.32	0.069	0.921	0.536	0.273
*SMCT*	0.033	0.001	0.005	0.002	0.002	0.005	0.002	0.002	0.002	0.001	0.002	0.0080	<0.001	0.007	<0.001
Bile acid receptor															
*FXR*	0.30	0.11	0.16	0.12	0.19	0.11	0.08	0.10	0.16	0.05	0.12	0.043	<0.001	0.080	0.581
Pattern recognition receptors															
*TLR1*	0.36	0.31	0.37	0.36	0.44	0.43	0.41	0.34	0.33	0.21	0.23	0.056	<0.001	0.588	0.695
*TLR2*	0.61	0.53	0.48	0.39	0.40	0.39	0.40	0.46	0.38	0.28	0.42	0.094	0.002	0.967	0.665
*TLR4*	0.38	0.43	0.39	0.44	0.54	0.55	0.61	0.51	0.61	0.47	0.45	0.069	<0.001	0.604	0.188
*TLR9*	0.39	0.32	0.34	0.35	0.38	0.34	0.40	0.35	0.42	0.34	0.35	0.066	0.759	0.298	0.967
Antimicrobial secretion															
*IAP*	0.0019	0.0001	0.0004	0.0002	0.0001	0.0003	0.0001	0.0001	0.0000	0.0000	0.0001	0.00062	<0.001	0.938	0.993
*MUC2*	0.33	0.42	0.47	0.42	0.50	0.47	0.47	0.46	0.44	0.43	0.43	0.086	0.076	0.841	0.795
*MUC4*	0.02	0.03	0.03	0.05	0.06	0.06	0.06	0.08	0.12	0.06	0.05	0.021	<0.001	0.494	0.556
Tight junction proteins															
*CLDN1*	0.39	0.34	0.35	0.36	0.35	0.32	0.31	0.22	0.20	0.14	0.11	0.065	<0.001	0.311	0.934
*CLDN4*	0.39	0.39	0.37	0.29	0.26	0.26	0.21	0.30	0.24	0.28	0.21	0.046	<0.001	0.068	0.740
*OCLN*	0.49	0.40	0.40	0.40	0.41	0.33	0.26	0.28	0.28	0.18	0.20	0.055	<0.001	0.883	0.878
*ZO1*	0.45	0.38	0.38	0.39	0.45	0.34	0.27	0.32	0.26	0.19	0.24	0.058	<0.001	0.967	0.432

Values are presented as least squares means ± SEM. *TLR1*, toll-like receptor 1; *TLR2*, toll-like receptor 2; *TLR4*, toll-like receptor 4; *TLR9*, toll-like receptor 9; *IAP*, intestinal alkaline phosphatase; *MUC2*, mucin 2; *MUC4*, mucin 4; *CLDN1*, claudin 1; *CLDN4*, claudin 4; *OCLN*, occludin; *ZO1*, zonula occludens-1. Day of life effect *P* < 0.05. At each time point, 10 piglets (5 male piglets and 5 female piglets) per dietary group were sampled.

### Relationship of lactate and short-chain fatty acid concentrations with relative expression of genes related to fatty acid recognition and transport

**Figure 3:**
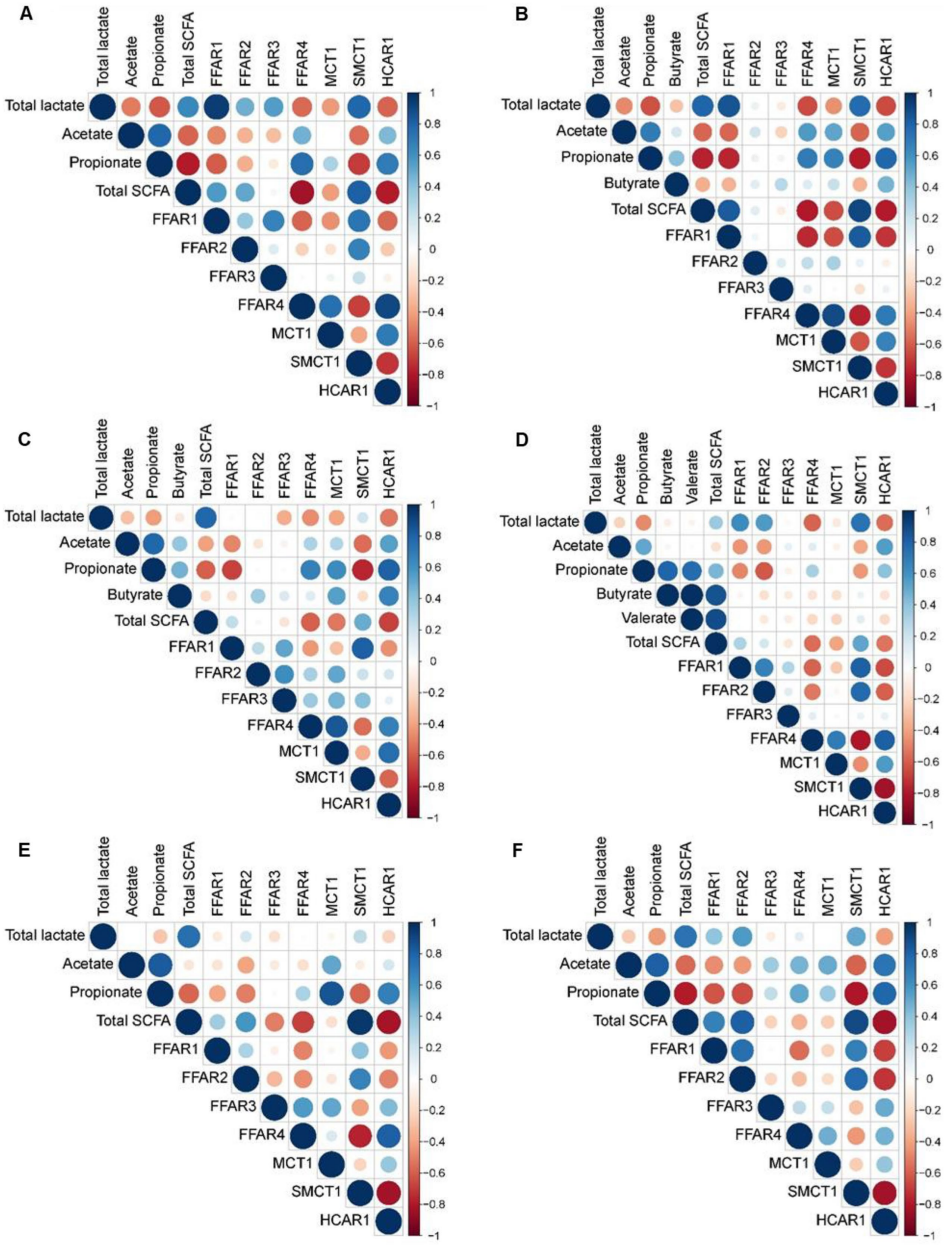
Correlation heat map using hierarchical cluster analysis showing associations of total lactate and SCFA concentrations in gastric digesta with relative expression levels of fatty acid receptors and transporters at the jejunal mucosa at day of life **(A)** 7, **(B)** 14, **(C)** 21, **(D)** 28, **(E)** 31, and **(F)** 35 *FFAR1*, free fatty acid receptor 1; *FFAR2*, free fatty acid receptor 2; *FFAR3*, free fatty acid receptor 3; *FFAR4*, free fatty acid receptor 4; *HCAR1*, hydroxycarboxylic acid receptor 1; *MCT1*, monocarboxylate transporter 1; *SMCT1*, sodium-coupled monocarboxylate transporter-1. At each time point, 10 piglets (5 male piglets and 5 female piglets) per dietary group were sampled.

**Figure 4:**
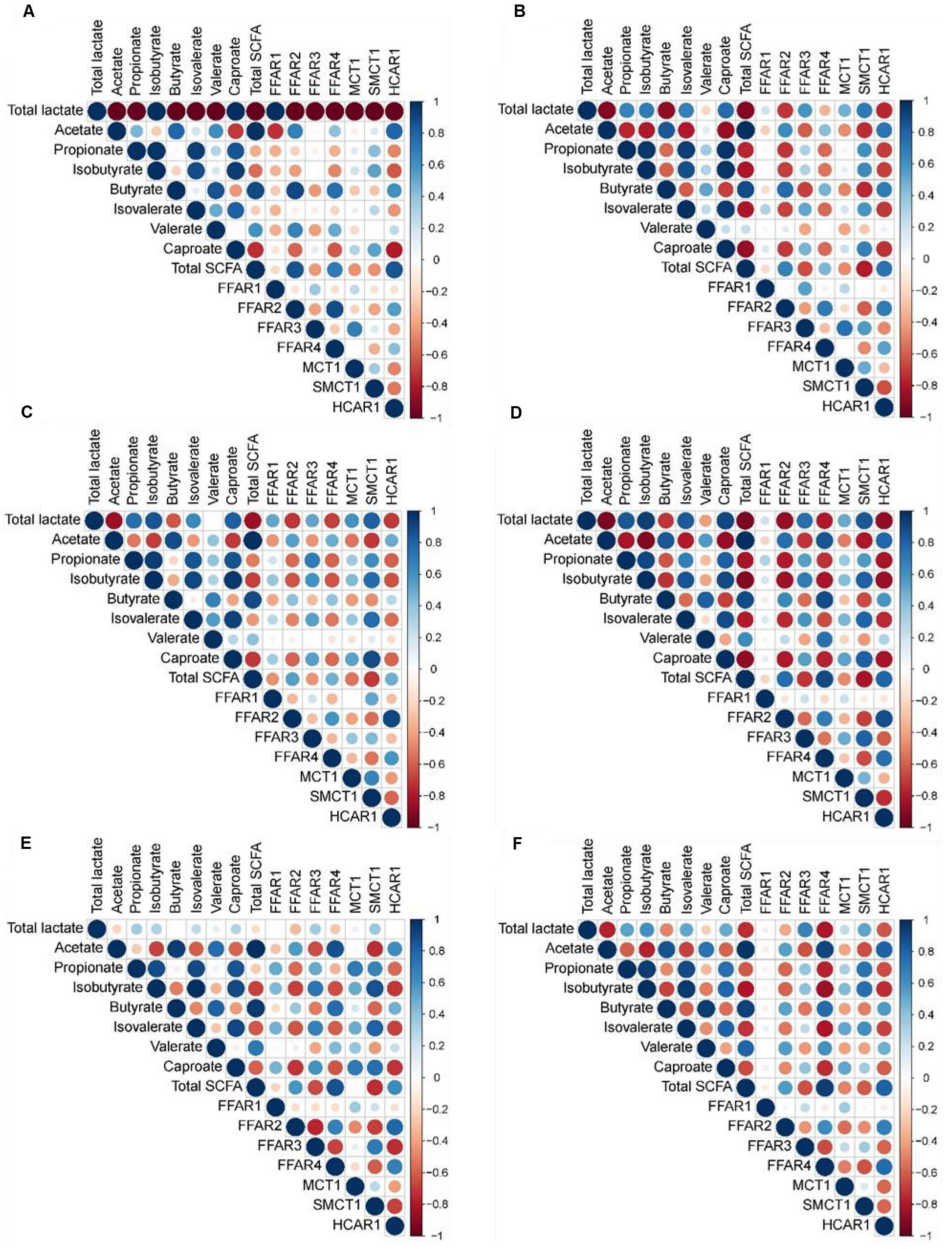
Correlation heat map using hierarchical cluster analysis showing associations of total lactate and SCFA concentrations in cecal digesta with relative expression levels of fatty acid receptors and transporters at the cecal mucosa at day of life **(A)** 7, **(B)** 14, **(C)** 21, **(D)** 28, **(E)** 31, and **(F)** 35. *FFAR1*, free fatty acid receptor 1; *FFAR2*, free fatty acid receptor 2; *FFAR3*, free fatty acid receptor 3; *FFAR4*, free fatty acid receptor 4; *HCAR1*, hydroxycarboxylic acid receptor 1; *MCT1*, monocarboxylate transporter 1; *SMCT1*, sodium-coupled monocarboxylate transporter 1. At each time point, 10 piglets (5 male piglets and 5 female piglets) per dietary group were sampled.

Pearson’s correlation coefficients revealed strong correlations between the concentration of lactate and SCFA in the stomach with expression of genes related to FA recognition and transport in the jejunum (|r| ≥ 0.75; Figure 3), as well as between cecal metabolite concentrations and gene expression (|r| ≥ 0.75; Figure 4). In the jejunum, expression levels of *FFAR1* and *SMCT1* positively correlated with gastric lactate concentration at DoL 7 and 14, and with total SCFA at DoL 14 and at DoL 7, 14, 31, and 35, respectively (0.76 < *r* < 0.96; Figure 3). Gastric propionate concentration negatively correlated with jejunal *FFAR1* expression at DoL 14 and with *SMCT1* expression at DoL 14, 21, and 35 (−0.80 < *r* < −0.76). Total gastric SCFA concentration positively correlated with jejunal *FFAR2* expression at DoL 35 (*r* = 0.82), whereas total SCFA negatively correlated with the expression of *FFAR4* at DoL 7 and 14, as well as with *HCAR1* at DoL 7, 14, 31, and 35 (−0.84 < *r* < −0.78). Propionate concentration in the stomach was positively associated with the expression levels of *FFAR4* at DoL 7, with *HCAR1* at DoL 14, 21, and 35, and with *MCT1* at DoL 31 in the jejunum (0.75 < *r* < 0.85). For the cecum, positive correlations of concentrations of acetate at DoL 28, butyrate at DoL 7 and 14, and total SCFA at DoL 7 and 28 with *FFAR2* expression were observed (0.75 < *r* < 0.90; Figure 4). Additionally, acetate at DoL 7, 28, and 35 and total SCFA at DoL 7, 28, and 35 positively correlated with *HCAR1* expression (0.78 < *r* < 0.85). The cecal concentration of lactate, propionate, isobutyrate, isovalerate, and caproate at DoL 28 negatively correlated with *FFAR2* and *HCAR1* expression. In addition, lactate at DoL 14 and caproate at DoL 7 negatively correlated with *HCAR1* expression (−0.88 < *r* < −0.75). A positive correlation was observed between cecal acetate, butyrate, and total SCFA concentration and *FFAR4* expression at DoL 28, 31, and 35, and additionally of butyrate with *FFAR4* expression at DoL 7 (0.75 < *r* < 0.93). By contrast, cecal lactate and isobutyrate at DoL 28 and 35, propionate and isovalerate at DoL 35, and caproate at DoL 28 negatively correlated with *FFAR4* expression (−0.86 < *r* < −0.77). Cecal concentrations of lactate, isobutyrate, isovalerate, and caproate were positively associated with *SMCT1* expression on DoL 21 and 31 (0.76 < *r* < 0.88). Additionally, cecal isobutyrate at DoL 31 and 35, as well as isovalerate and caproate at DoL 31, positively correlated with *SMCT1* expression (0.75 < *r* < 0.88). By contrast, acetate at DoL 28, butyrate at DoL 14, and total SCFA concentration at DoL 14 and 28 were negatively associated with *SMCT1* expression at the cecal mucosa (−0.81 < *r* < −0.75).

## Discussion

The present study provides novel insights into age-related dynamics and the impact of creep feeding in the microbe-host interplay in suckling and early-weaned piglets. The effects of creep feeding on the investigated parameters were rather small, which may be related to the low intake at the beginning and the importance of sow milk for gut development during the suckling phase. This study provides novel data for the neonatal microbiome development in the stomach and cecum, gut sites for which little data were available so far. Our results for the gastric total microbial abundances demonstrated that microbial numbers increased throughout the suckling phase. From the composition, the dominance of *Lactobacillaceae* and higher abundances of *Streptococcaceae* and *Pasteurellaceae* in gastric digesta throughout the suckling phase may be explained by their capacity to ferment milk glycans, such as lactose and oligosaccharides (4, 30, 31). Similar to the bacterial composition, the concentrations of fermentation acids (i.e., lactate and acetate) and pH remained stable during the suckling period, which may be related to the retention time of the feed in the stomach and end-product inhibition. The produced fermentation acids, such as lactate and acetate, are important for compensating for the low stomach acid production in neonatal piglets (32, 33). Opposite to the stomach, cecal microbial numbers (except for protozoa) were stable throughout the suckling phase, whereas the compositional changes (bacteria and fungi) and increase in SCFA were greater in the cecal digesta compared to gastric digesta from DoL 7 to 28. *Bacteroidaceae* predominated the cecal bacterial community at DoL 7, which can be related to their capability to ferment milk-glycans, and its predominance is in accordance with previous data for feces of suckling piglets (4, 9, 34). The appearance of *Prevotellaceae* in the feces of neonatal piglets was often linked to the availability of plant carbohydrates (4, 9, 34). However, the present abundance of *Prevotellaceae* of >20% in cecal digesta throughout the suckling phase demonstrates their metabolic flexibility to thrive on different substrates, including milk components, host secretions, and probably primary fermentation metabolites (2, 35). The latter likely also promoted other predominant families in the cecal community. For instance, members within *Clostridiaceae*, *Peptostreptococcaceae*, and *Erysipelotrichaceae* can utilize host mucin (36). *Lachnospiraceae*, another dominant family, can also use a variety of glycans including dietary glycans and mucus (37). Whether the abundance of these taxa was characteristic of the microbiome at our farm (10) or describes ubiquitous fluctuations in the cecal microbiome of suckling piglets needs further investigation. Total protozoa in cecal digesta showed a similar development to that in the stomach; novel findings that also need further investigation and may be explained by gastrointestinal conditions, and microbe-microbe, milk-microbe, or host–microbe interactions. Intestinal colonization with fungi increases with the intake of plant material as a major substrate source (13, 38). In this study, we focused on the cecum as a major ‘fermentation chamber’ and on four time points that were selected based on the results for the absolute fungal abundances. Our findings for the age-related fungal development in gastric and cecal digesta emphasize the importance to investigate the role of fungal development in the host’s upper and lower gut from birth to understand how it affects the host. The changes in the dominant yeast families *Dipodascaceae* and *Saccharomycetaceae* and the ascomycete family *Didymellaceae* from DoL 14–28 may be explained by microbe-fungal interactions and uptake from the environment [e.g., floor, sow feces or diet; (13, 38)]. A certain role of milk components may be also thinkable in this interplay but needs further investigation.

The host’s maturational processes follow a genetic program after birth, which is influenced by the local gut microbiota (15). The current positive and negative Pearson correlations (jejunum and cecum) and associations from the sPLS-DA (cecum) would support that microbes, i.e., bacteria and fungi, and microbial metabolites influence the expression of receptors for SCFA, BA, and microbial surface structures as well as of SCFA and medium-chain fatty acid transporters. The present developmental patterns of the jejunal expression of the four investigated TLRs from DoL 7 to 28 differed from that reported by Arnaud et al. (15), suggesting the importance of the actual microbial composition at the gut site for TLR expression. In our study, the initial gut microbiota composition seemed to have triggered a stronger jejunal response of TLR-1, −2, −4, and −9 on DoL 7 compared to the study of Arnaud et al. (15). These TLRs are activated by bacterial ligands, such as lipoproteins, lipopolysaccharides, and DNA (39). The declining jejunal TLR expression from DoL 7–28 may be indicative of the build-up of immune unresponsiveness toward the luminal microbiome (2, 40). Due to differences in the microbiome composition, the cecal TLR expression likely followed a different developmental pattern compared to the jejunum. Expression levels of tight-junction proteins, mucins, and *IAP* in the jejunum and cecum showed a developmental pattern like the TLR expression. Its expression may have been triggered via the activation of TLRs and subsequent cytokine production (41, 42).

Similar to the age-related changes for the receptor expression, we observed a shortening of jejunal villi and the absorptive surface from DoL 7–28; findings that may reflect the high nutrient demand of the piglet in the immediate time after birth (17). At both gut sites, the secretory processes and cell renewal potential increased as indicated by the deepening of the crypts (43, 44). According to the higher expression of *FXR* at the jejunal and cecal mucosa on DoL 7 compared to the other DoLs, BA signaling may play an important role in the developing gut, which needs further investigation.

The low feed intake, a complete change to the pre-starter diet, and the lack of sow milk resulted in drastic alterations of the bacterial (stomach and cecum) and fungal communities (cecum) and led to the macroscopically visible inflammation from the esophagus to the rectum on DoL 31. Fungal abundance increased in gastric digesta from DoL 28–31, potentially due to a combined effect of higher fungal uptake with the feed and change in factors controlling the growth of fungi (e.g., bacteria or milk compounds). After weaning, *Erysiphaceae* (also known as powdery mildews) made up more than half of the fungal community. As plant pathogens (45), their origin probably was the plant-based feed and/or the straw bedding. The sPLS-DA supported that fungi, such as *Aspergillus* belonging to the moderately abundant *Trichocomaceae*, have a role in the microbe-host interplay (13).

The weaning-associated bacterial alterations were mainly compositional changes, whereas the total bacterial numbers remained stable from DoL 28–35. This may indicate that microbial niches were taken over by other taxa that could rely on host secretions, such as mucus, and/or utilize plant-based carbohydrates. From DoL 28–31, *Lactobacillaceae* largely dropped in gastric digesta, which were replaced by the pathobionts *Rickettsiales* and *Pasteurellaceae* (46, 47). The same was true for the cecum where *Bacteroidaceae* disappeared and seemed to be replaced by hemicellulolytic and mucin-degrading *Lachnospiraceae* on DoL 31 and 35. Host effects, including the macroscopically visible inflammation, were presumably also caused by a combination of the lack of luminal nutrition from the diet, lack of sow milk, microbial changes, and low SCFA after weaning (41–44). In fact, luminal SCFA and medium-chain fatty acids can suppress virulence factor expression in pathobionts and moderate the local TLR response (41–44). The detrimental impact of weaning on the gut epithelium was more visible at structural than at the gene expression level and characterized by blunting of jejunal villi and the shallowing of crypts in the jejunum and cecum from DoL 28 to DoL 31. By contrast, expression levels of *FFAR2*, *FFAR3*, and *FXR*, which may mediate anti-inflammatory signals, were unchanged on DoL 31 compared to DoL 28. Likewise, expression levels of the four investigated TLRs did not show a weaning-associated upregulation on DoL 31, which may indicate that either the sampling timepoint was too late, other pattern-recognition receptors were activated, or the activation was only visible at translational and hence functional protein levels. The first assumption would be supported by recent observations from our group using the intestinal loop perfusion assay that the milk-primed gut mucosa (jejunum and colon) reacts to postweaning digesta with an upregulation of the innate immune response within 2 h of exposure (25). Deeper crypts, recruitment of goblet cells, expression of *MUC4* (only cecum), and intraepithelial lymphocytes post-weaning at both gut sites indicated increased secretory processes, supporting the upregulation of an innate immune response (15). Interestingly, jejunal *OCLN* expression re-increased at DoL 35 compared to the pre-weaning level but not in the cecum, which may imply a faster restoration of mucosal barrier function in the small intestine compared to the large intestine.

The creep feed intake of individual piglets is unpredictable and often low (48). Accordingly, the estimated daily creep feed intake was low at the beginning, but in the range reported previously (48). It gradually increased to amounts that substantially contributed to the nutrition of the piglet in the fourth week of life. Nevertheless, the few feeding effects and the missing DoL×feeding interactions indicated that the creep feeding influenced the developmental patterns in the investigated gut segments less than expected, suggesting a strong influence of porcine milk components for the gut microbial-host developmental interplay. A reason for our observations may be the composition of the offered milk replacer, which contained a high amount of bovine whey powder (43% DM). Whey is rich in milk glycans (49), but the oligosaccharide profile and lactose content in bovine milk are different from that of porcine milk (49), possibly reducing the concentrations of acetate (DoL 14 and 21) and lactate (DoL 21) in gastric digesta. This change in luminal fatty acid concentrations may have altered microbe-microbe interactions and mucosal signaling in the stomach and upper small intestine. The lack of a change in jejunal expression levels for FFARs, *HCAR1*, and monocarboxylate transporters led us to assume that microbial fatty acids were similar in this segment. However, also after the switch to the starchy and more fibrous pre-starter diet, microbial abundances and fermentation seemed to be mostly driven by porcine milk on DoL 28. In cecal digesta, the starch fraction in the creep feed, which was similar in both types of feed, presumably raised starch-degrading *Prevotellaceae* and *Ruminococcaceae* from DoL 14 but without changing the concentrations of fermentation end-products and altering mucosal expression of the respective receptors, transporters or TLRs. *Prevotellaceae* and *Ruminococcaceae* also contain hemicellulolytic species (4, 9, 34), potentially enabling the creep-fed piglets to better utilize the fiber fraction after weaning on DoL 31 and 35. The PLS-DA identified mainly lower abundant genera in the cecal digesta of the two piglet groups as discriminative, supporting hemicellulolytic, milk glycan-degrading, and cross-feeding capabilities. Nevertheless, it should be mentioned that due to their low abundance, it can be speculated how strongly these taxa influenced host physiology. Of note, the creep-feeding-induced bacterial differences in cecal digesta persisted and were still detectable after weaning on DoL 35, which may give these piglets an advantage in the fermentation of dietary fiber and production of anti-inflammatory SCFA. By contrast, their uptake with the pre-starter diet likely explains the higher abundance of *Didymellaceae* in the cecal fungal community in creep-fed piglets on DoL 28. The sPLS-DA identified discriminative genes for the suckling and postweaning phase for the two piglet groups including fatty acid transporters and receptors. However, these data rather describe a trend and should not be overinterpreted as the differential statistical analysis showed no difference for the expressed genes.

In conclusion, our results provide detailed insights into the age- and gut-site-related alterations in microbial taxonomic and metabolite composition in gastric and cecal digesta and host mucosal development in the jejunum and cecum. The age-dependent alterations, partly genetically programmed and caused by the continuously changing gut microbiome, had a strong impact on the expression of genes for gut barrier function, integrity, innate immunity, and SCFA signaling. Due to the low intake, creep feeding seemed to play a minor role in the temporal dynamics in the microbiome and mucosal response in the investigated gut segments in the present study. This study is one of the first studies proposing fungi as important modulators for the mucosal expression of genes involved in nutrient transport, immune response, and barrier function in neonatal piglets. Our observation may provide valuable information for the optimization of nutritional concepts to ease the weaning transition in piglets.

## Data availability statement

The datasets generated for this study can be found at the NCBI Bioproject databank (PRJNA907751).

## Ethics statement

All procedures involving animal handling and treatment were approved by the Institutional Ethics Committee of the University of Veterinary Medicine Vienna and the National authority according to the Law for Animal Experiments, Tierversuchsgesetz (BMWFW- 68.205/00936- V3b/2019). The pig experiment was performed in accordance with the university and national regulations.

## Author contributions

BM-Z conceived the study, acquired funding, applied for Ethical Committee approval, analyzed sequencing data, and edited and finalized the manuscript. BM-Z, FL, and FY conducted the experiment. BM-Z, FL, FY, JV, and SK collected the biological samples. SK, JE, and SS processed samples. BM-Z and FL analyzed the data and wrote the original manuscript draft. All authors read and approved the final version of the manuscript.

## Funding

This research was funded by the Austrian Federal Ministry for Digital and Economic Affairs and the National Foundation for Research, Technology and Development. The Christian Doppler Laboratory for Innovative Gut Health Concepts of Livestock is financially supported by BIOMIN Holding GmbH, which is part of DSM-Firmenich. The funder was not involved in the study design, analysis, interpretation of data, the writing of this article or the decision to submit it for publication.

## Conflict of interest

The authors declare that the research was conducted in the absence of any commercial or financial relationships that could be construed as a potential conflict of interest.

## Publisher’s note

All claims expressed in this article are solely those of the authors and do not necessarily represent those of their affiliated organizations, or those of the publisher, the editors and the reviewers. Any product that may be evaluated in this article, or claim that may be made by its manufacturer, is not guaranteed or endorsed by the publisher.

## Supplementary material

The Supplementary material for this article can be found online at: https://www.frontiersin.org/articles/10.3389/fvets.2023.1184277/full#supplementary-material

## Footnotes

^1^ https://cutadapt.readthedocs.org/

^2^ www.ncbi.nlm.nih.gov/tools/primer-blast/
