# Supplementary material for: An insight into the temporal dynamics in the gut microbiome, metabolite signaling, immune response, and barrier function in suckling and weaned piglets under production conditions
Source: Front Vet Sci. 2023 Aug 31;10:1184277. doi: 10.3389/fvets.2023.1184277 (PMC10500839; doi:10.3389/fvets.2023.1184277)
Supplement: Supplementary file 1 [file Data_Sheet_1.docx]

**Supplementary Material:**

**Table S1.** Analyzed nutrient composition of the piglets and lactation diet.

| **Chemical composition, % DM** | **Lactation diet^3^** | **Milk replacer^4^** | **Prestarter diet^5^** |
| --- | --- | --- | --- |
| Dry matter, % | 89.0 | 94.8 | 91.9 |
| Crude ash | 5.5 | 5.6 | 5.5 |
| Crude protein | 17.9 | 19.8 | 20.5 |
| Crude fiber | 5.8 | 0.4 | 5.2 |
| Neutral-detergent fibre | 17.3 | 3.0 | 15.2 |
| Acid-detergent fibre | 6.7 | 0.6 | 5.8 |
| Acid-detergent lignin | 1.8 | 0.3 | 1.5 |
| Crude fat | 5.2 | 7.8 | 7.5 |
| Nitrogen-free extract | 65.7 | 66.5 | 61.6 |
| Starch | 47.3 | 29.4 | 31.9 |
| Sugar | 5.5 | 26.8 | 14.2 |
| Metabolizable energy, MJ/kg | 14.7 | 16.9 | 15.3 |

^1^ZuchtsauenKorn S Vital, Garant-Tiernahrung GmbH, Pöchlarn, Austria. Ingredient composition: corn, wheat, barley, soybean meal, sunflower meal, wheat bran, apple pomace, soybean oil, calcium carbonate, monocalcium phosphate, sodium chloride, magnesium phosphate, fish oil, L-cellulose, molasses. Vitamin and mineral composition per kg feed: 10,000 IU of vitamin A, 1,800 IU of vitamin D, 100 mg of Fe as iron(II) sulfate, 15 mg of Cu as copper(II) sulfate, 90 mg of Zn as zinc sulfate, 40 mg of Mn as manganese(II) oxide, 1.5 mg of I as calcium iodate, 0.4 mg of Se as sodium selenite. Technological additives: 500 FTU phytase, 2 mg of butylated hydroxyanisole, 10 mg of butylated hydroxytoluene, 2 mg of propyl gallate.

^2^Weanplus-4, Startix, Voorthuizen, The Netherlands. Ingredient composition: Whey powder, starch, soy protein, plant protein, plant oil (coconut oil and palm oil). Vitamin and mineral composition per kg feed: 25,000 IU of vitamin A, 8,000 IU of vitamin D3, 200 mg of vitamin E, 140 mg of Cu as chelate of glycine hydrate, 108 mg of Fe as chelate of glycine hydrate, 3 mg of I as calcium iodate, 115 mg of Zn as chelate of glycine hydrate, 35 mg of Mn as glycine chelate, 0.3 mg of Se as sodium selenite. Technological additives: 5 g of citric acid, 1 g of calcium formiate. Mixing ratio: 200 g of powder mixed into 1 L of 45°C warm water.

^3^Ferkelabsetzkorn OGT, Garant-Tiernahrung GmbH, Pöchlarn, Austria. Ingredient composition: Oat flakes, barley, wheat, whey powder, soy protein concentrate, wheat bran, sucrose, soybean meal, soy oil, corn gluten, L-cellulose, monocalcium phosphate, fish oil, sodium chloride, magnesium phosphate, calcium carbonate and molasses. Vitamin and mineral composition per kg feed: 16,000 IU of vitamin A, 2,000 IU of vitamin D3, 200 mg vitamin E, 120 mg Fe as iron(II) sulfate, 140 mg Cu as copper(II) sulfate, 120 mg Zn as zinc sulfate, 60 mg Mn as manganese(II) oxide, 1.5 mg I as calcium iodate, 0.5 mg Se as sodium selenite. Technological additives: 1,000 IU of phytase, 1,500 EPU of xylanase, 11 mg of beta hydroxy acid, 21 mg of butylated hydroxytoluene, 11 mg of propyl gallate.

**Table S2.** Oligonucleotide primers for bacteria, protozoa, fungi and yeasts, as well as archaea used for quantitative PCR in gastric and cecal digesta of piglets receiving only sow milk or additionally creep feed.

| **Group** | **Primer sequence (5’ to 3’)^1^** | | **R^2^** | **Efficiency (%)** | **Amplicon size (bp)** | **Reference** |
| --- | --- | --- | --- | --- | --- | --- |
| Total bacteria | F: | CCTACGGGAGGCAGCAG | 0.999 | 93 | 193 | [1] |
|  | R: | ATTACCGCGGCTGCTGG |  |  |  |  |
| Total protozoa | F: | GCTTTCGWTGGTAGTGTATT | 0.998 | 92 | 233 | [2] |
|  | R: | CTTGCCCTCYAATCGTWCT |  |  |  |  |
| Total fungi and yeasts | F: | GCATATCAATAAGCGGAGGAAAAG | 0.997 | 93 | 250 | [3] |
|  | R: | ATTCCCAAACAACTCGACTC |  |  |  |  |
| Total archaea | F: | CCGGAGATGGAACCTGAGAC | 0.997 | 98 | 160 | [4] |
|  | R: | CGGTCTTGCCCAGCTCTTATTC |  |  |  |  |

^1^F, forward primer; R, reverse primer.

**Table S3.** Oligonucleotide primers used for quantitative PCR to assess jejunal and cecal gene expression of piglets receiving only sow milk or additionally creep feed from day 10 of life.

| **Genes^1^** | **Accession number^2^** | **Primer sequence (5’ to 3’)^3^** | | **R^2^** | **Efficiency (%)** | **Amplicon size (bp)** | **Reference** |
| --- | --- | --- | --- | --- | --- | --- | --- |
| **Reference genes** | | | | | | | |
| *ACTG* | XM_003357928.4 | F: | GGGCATCCTGACCCTCAAG | 1.000 | 99 | 89 | [5] |
|  |  | R: | TGTAGAAGGTGTGATGCCAGATCT |  |  |  |  |
| *B2M* | NM_213978.1 | F: | CCCCCGAAGGTTCAGGTT | 1.000 | 98 | 66 | [6] |
|  |  | R: | GCAGTTCAGGTAATTTGGCTTTC |  |  |  |  |
| *GAPDH* | NM_001206359.1 | F: | GGCGTGAACCATGAGAAGTATG | 0.999 | 99 | 60 | [6] |
|  |  | R: | GGTGCAGGAGGCATTGCT |  |  |  |  |
| *HPRT* | NM_001032376.2 | F: | AGAAAAGTAAGCAGTCAGTTTCATATCAGT | 0.999 | 90 | 131 | [6] |
|  |  | R: | ATCTGAACAAGAGAGAAAATACAGTCAATAG |  |  |  |  |
| *OAZ1* | NM_001122994.2 | F: | TCGGCTGAATGTAACAGAGGAA | 0.999 | 99 | 70 | [6] |
|  |  | R: | GAGCCTGGATTGGACGTTTAAA |  |  |  |  |
| **Fatty acid signalling and transport** | | | | | | | |
| *FFAR1* | XM_013998289.2 | F: | ACTTAGGGAAAGAACTGAGCCT | 0.998 | 95 | 105 | Newly designed |
|  |  | R: | GATGAAATGCGGCAGCTTACC |  |  |  |  |
| *FFAR2* | NM_001278758.1 | F: | CTGCCTGGGATCGTCTGTG | 1.000 | 100 | 249 | [7] |
|  |  | R: | CATACCCTCGGCCTTCTGG |  |  |  |  |
| *FFAR3* | NM_001315601.1 | F: | GCCCTTGCCCTTCATCTTCT | 0.999 | 99 | 136 | [7] |
|  |  | R: | CCGGGTCTTGTACCAGAGTG |  |  |  |  |
| *FFAR4* | NM_001204766.2 | F: | GCACCCGTGTACCTGCTTTA | 0.998 | 100 | 127 | Newly designed |
|  |  | R: | AAGGAACCCACAGCAAATCCTTT |  |  |  |  |
| *HCAR1* | NM_001145381.1 | F: | AATGCCATCTCCAACCGGAC | 1.000 | 96 | 153 | Newly designed |
|  |  | R: | GCCATTGGCTGACTCCATGA |  |  |  |  |
| *MCT1* | AM286425.1 | F: | GGTGGAGGTCCTATCAGCAG | 0.998 | 95 | 74 | [8] |
|  |  | R: | AAGCAGCCGCCAATAATCAT |  |  |  |  |
| *SMCT1* | NM_001291414.1 | F: | AATCCTCACCTGCTCAGTGC | 1.000 | 98 | 172 | [9] |
|  |  | R: | GTAAGCGCAGGCCACAAAAA |  |  |  |  |
| **Bile acid receptor** | | | | | | | |
| *FXR* | KF597010.1 | F: | AAGCCTGCCAAAGGTGTACT | 1.000 | 94 | 156 | Newly designed |
|  |  | R: | GGGGTAGAAACCCAGGTTGG |  |  |  |  |
| **Pattern recognition receptors** | | | | | | | |
| *TLR1* | AB086376.1, NM_001031775.1 | F: | TTTGCCCACCACAACCTCTT | 0.999 | 99 | 153 | Newly designed |
|  |  | R: | GCTCTTCTCCTTGGGCCATT |  |  |  |  |
| *TLR2* | NM_213761.1 | F: | AATAAGTTGAAGACGCTCCCAGAT | 0.999 | 94 | 97 | [8] |
|  |  | R: | GTTGCTCCTTAGAGAAAGTATTGATCGT |  |  |  |  |
| *TLR4* | AB188301.2 | F: | TGTGGCCATCGCTGCTAAC | 0.998 | 99 | 124 | [8] |
|  |  | R: | GGTCTGGGCAATCTCATACTCA |  |  |  |  |
| *TLR9* | AY859728 | F: | CACGACAGCCGAATAGCAC | 0.998 | 94 | 122 | [10] |
|  |  | R: | GGGAACAGGGAGCAGAGC |  |  |  |  |
| **Antimicrobial secretion** | | | | | | | |
| *IAP* | XM_003133729.4 | F: | AGGAACCCAGAGGGACCATTC | 1.000 | 94 | 83 | [8] |
|  |  | R: | CACAGTGGCTGAGGGACTTAGG |  |  |  |  |
| *MUC2* | XM_021082584.1 | F: | GCTCCAGAGAGAAGGCAGAA | 0.999 | 94 | 162 | Newly designed |
|  |  | R: | ACAGCGAACTCCTTGTAGGC |  |  |  |  |
| *MUC4* | DQ848681.1 | F: | GAGCAGAGCCCTGAGGGTA | 1.000 | 93 | 101 | Newly designed |
|  |  | R: | CCCTGGAACCAGAGCTTCAG |  |  |  |  |
| **Tight junction proteins** | | | | | | | |
| *CLDN1* | NM_001244539.1 | F: | TGATGAGGTGCAGAAGATGC | 1.000 | 96 | 88 | [5] |
|  |  | R: | CCATGCTGTGGCAACTAAGA |  |  |  |  |
| *CLDN4* | NM_001161637.1 | F: | CAACTGCGTGGATGATGAGA | 0.995 | 105 | 140 | [5] |
|  |  | R: | CCAGGGGATTGTAGAAGTCG |  |  |  |  |
| *OCLN* | NM_001163647.2 | F: | TTGTGGGACAAGGAACGTATTTA | 1.000 | 97 | 76 | [5] |
|  |  | R: | TGCCTGCCGACACGTTT |  |  |  |  |
| *ZO1* | AJ318101.1 | F: | TCAAGGTCTGCCGAGACAAC | 0.999 | 100 | 75 | [9] |
|  |  | R: | CCAAAGGACTCAGCAGGGTT |  |  |  |  |

^1^*ACTG*, γ-actin; *B2M*, β2-microglobulin; *GAPDH*, glyceraldehyde-3-phosphate-dehydrogenase; *HPRT*, hypoxanthin-guanine phosphoribosyl transferase; *OAZ1*, ornithine decarboxylase antizyme 1; *FFAR1*, free fatty acid receptor 1; *FFAR2*, free fatty acid receptor 2; *FFAR3*, free fatty acid receptor 3; *FFAR4*, free fatty acid receptor 4; *HCAR1*, hydroxycarboxylic acid receptor 1; *MCT1*, monocarboxylate transporter 1; *SMCT1*, sodium coupled monocarboxylate transporter 1; *FXR*, farnesoid X receptor; *TLR1*, toll-like receptor 1; *TLR2*, toll-like receptor 2; *TLR4*, toll-like receptor 4; *TLR9*, toll-like receptor 9; *IAP*, intestinal alkaline phosphatase; *MUC2*, mucin 2; *MUC4*, mucin 4; *CLDN1*, claudin 1; *CLDN4*, claudin 4; *OCLN*, occludin; *ZO1*, zonula occludens-1.

^2^National Center for Biotechnology Information (NCBI; http://www.ncbi.nlm.nih.gov/sites/entrez?db=gene).

^3^F, forward primer; R, reverse primer.

**Table S4.** Descriptive statistics for average daily creep feed intake during the suckling period.

| Daily intake (g dry matter) | Mean | SE | Minimum | Maximum | Median |
| --- | --- | --- | --- | --- | --- |
| DoL 10-16 | 10 | 1.9 | 4 | 23 | 8 |
| DoL 17-23 | 18 | 3.7 | 7 | 41 | 14 |
| DoL 24-25 | 30 | 3.8 | 14 | 52 | 29 |
| DoL 26-28 | 79 | 10.3 | 40 | 125 | 78 |

Creep feed consumption was measured at litter level. SE, standard error of the mean.

**Table S5.** Body weight development and average daily gain from birth until day of life (DoL) 34 of piglets receiving only sow milk or additionally receiving creep feed from DoL10.

| **Feed** | **Sow milk** | **Creep feed** | **SEM** | **P value^1^** |
| --- | --- | --- | --- | --- |
|  |  |  |  | **Diet** |
| **Body weight, kg** | | | | |
| DoL1 | 1.58 |  | 0.034 | . |
| DoL2 | 1.79 |  | 0.044 | . |
| DoL6 | 2.35 |  | 0.064 | . |
| DoL13 | 3.98 | 3.83 | 0.134 | 0.496 |
| DoL20 | 5.57 | 5.64 | 0.134 | 0.824 |
| DoL27 | 7.51 | 7.43 | 0.208 | 0.815 |
| DoL30 | 7.51 | 7.52 | 0.226 | 0.980 |
| DoL34 | 9.02 | 7.23 | 0.284 | 0.005 |
| **Average daily gain, kg/d** | | | | |
| DoL1 to 6 | 0.12 |  | 0.006 | 0.720 |
| DoL6 to 13 | 0.22 |  | 0.012 | 0.285 |
| DoL13 to 20 | 0.24 | 0.27 | 0.013 | 0.159 |
| DoL20 to 27 | 0.25 | 0.25 | 0.009 | 0.856 |
| DoL27 to 30 | 0.04 | 0.04 | 0.015 | 0.977 |
| DoL30 to 34 | 0.02 | 0.11 | 0.040 | 0.205 |
| DoL13 to 27 | 0.24 | 0.26 | 0.011 | 0.357 |
| DoL13 to 34 | 0.19 | 0.18 | 0.019 | 0.774 |
| DoL27 to 34 | 0.04 | 0.06 | 0.036 | 0.716 |

Values are presented as least squares means ± SEM.

^1^Fixed effect of day of life was significant for all parameters in the repeated model (P < 0.05).

**Table S6.** Age-related development of gastric and cecal size per kg body weight in piglets receiving only sow milk or additionally creep feed from day of life (DoL) 10.

| **Feed** | **Sow milk** | **Creep feed** | **SEM** | **P value^1^** |
| --- | --- | --- | --- | --- |
|  |  |  |  | **Diet** |
| **Stomach, cm/kg body weight** | | | | |
| DoL7 | 3.20 |  | 0.173 | . |
| DoL14 | 1.71 | 1.92 | 0.100 | 0.151 |
| DoL21 | 1.43 | 1.62 | 0.081 | 0.113 |
| DoL28 | 1.34 | 1.45 | 0.102 | 0.461 |
| DoL31 | 1.40 | 1.46 | 0.096 | 0.703 |
| DoL35 | 1.63 | 1.71 | 0.093 | 0.597 |
| **Cecum, cm/kg body weight** | | | | |
| DoL7 | 1.90 |  | 0.092 | . |
| DoL14 | 1.50 | 1.31 | 0.104 | 0.220 |
| DoL21 | 1.12 | 1.31 | 0.071 | 0.086 |
| DoL28 | 1.05 | 1.14 | 0.082 | 0.455 |
| DoL31 | 1.32 | 1.47 | 0.093 | 0.277 |
| DoL35 | 1.25 | 1.29 | 0.112 | 0.802 |

Values are presented as least squares means ± SEM.

^1^Fixed effect of day of life was significant for all parameters in the repeated model (P < 0.05).

**Figure S1.** Age-related development in species richness (Chao1) and alpha diversity (Shannon and Simpson) indices for the bacteriome in (**A-C**) gastric and (**D-F**) cecal digesta, and (**G-I)** for the mycobiome in cecal digesta of piglets fed sow milk only or receiving additional creep feed from day of life 10.


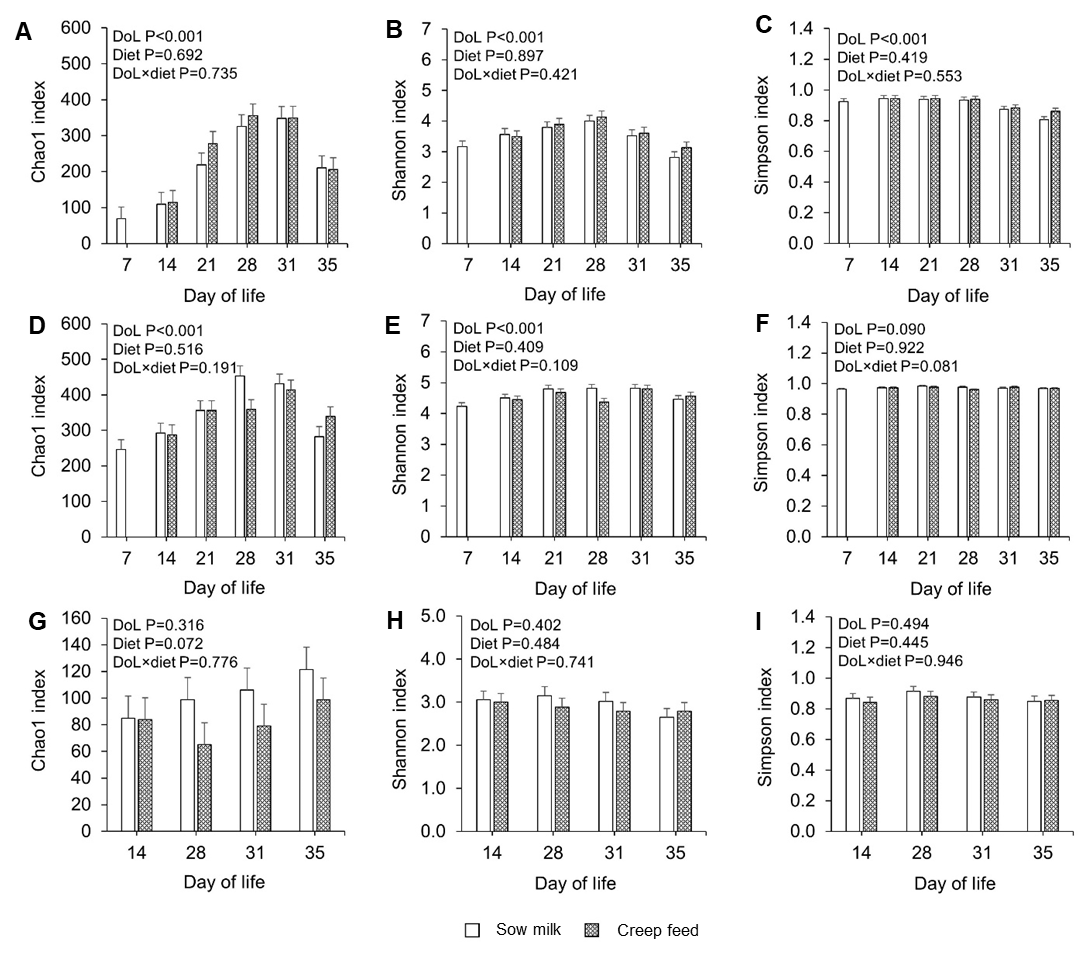


**Figure S2.** Loading plots of sparse partial square-discriminant analysis displaying the most discriminant genes expressed at the mucosa and fungal and bacterial genera in the cecum of piglets receiving only sow milk (blue) or additionally creep feed from day of life 10 (orange) at day of life (**A**) 14, (**B**) 21, (**C**) 28, (**D**) 31, and (**E**) 35. *FFAR1*, free fatty acid receptor 1; *FFAR3*, free fatty acid receptor 3; *FFAR4*, free fatty acid receptor 4; *HCAR1*, hydroxycarboxylic acid receptor 1; *MCT1*, monocarboxylate transporter 1; *SMCT1*, sodium coupled monocarboxylate transporter 1; *FXR*, farnesoid X receptor; *TLR1*, toll-like receptor 1; *TLR2*, toll-like receptor 2; *TLR4*, toll-like receptor 4; *TLR9*, toll-like receptor 9; *IAP*, intestinal alkaline phosphatase; *MUC4*, mucin 4; *CLDN1*, claudin 1; *CLDN4*, claudin 4; *OCLN*, occludin; *ZO1*, zonula occludens-1.


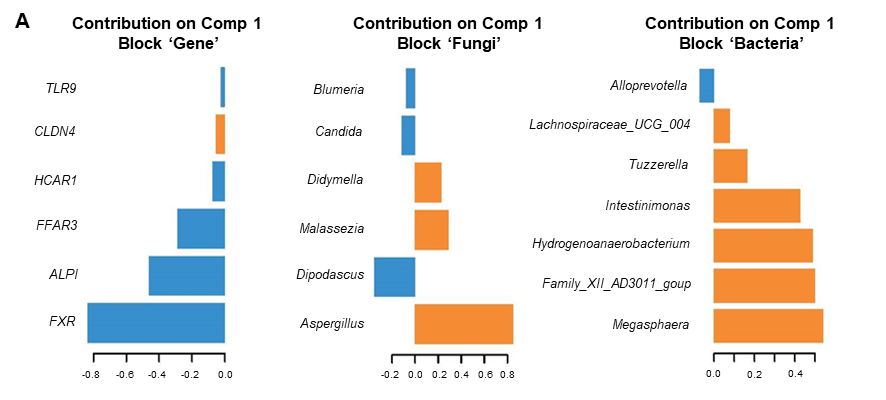


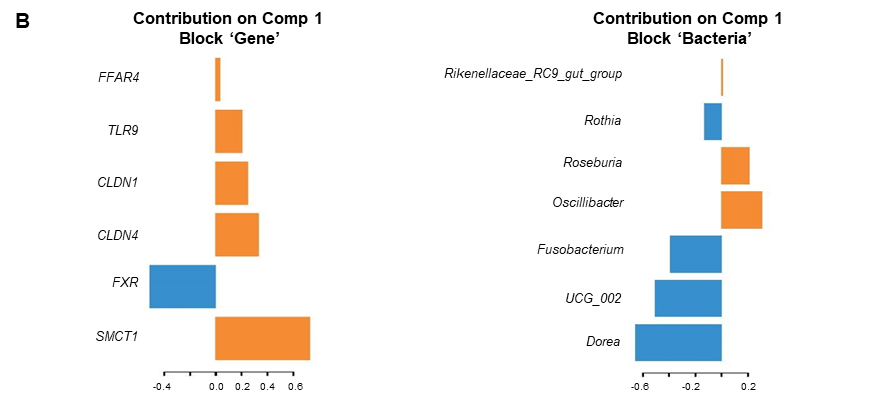


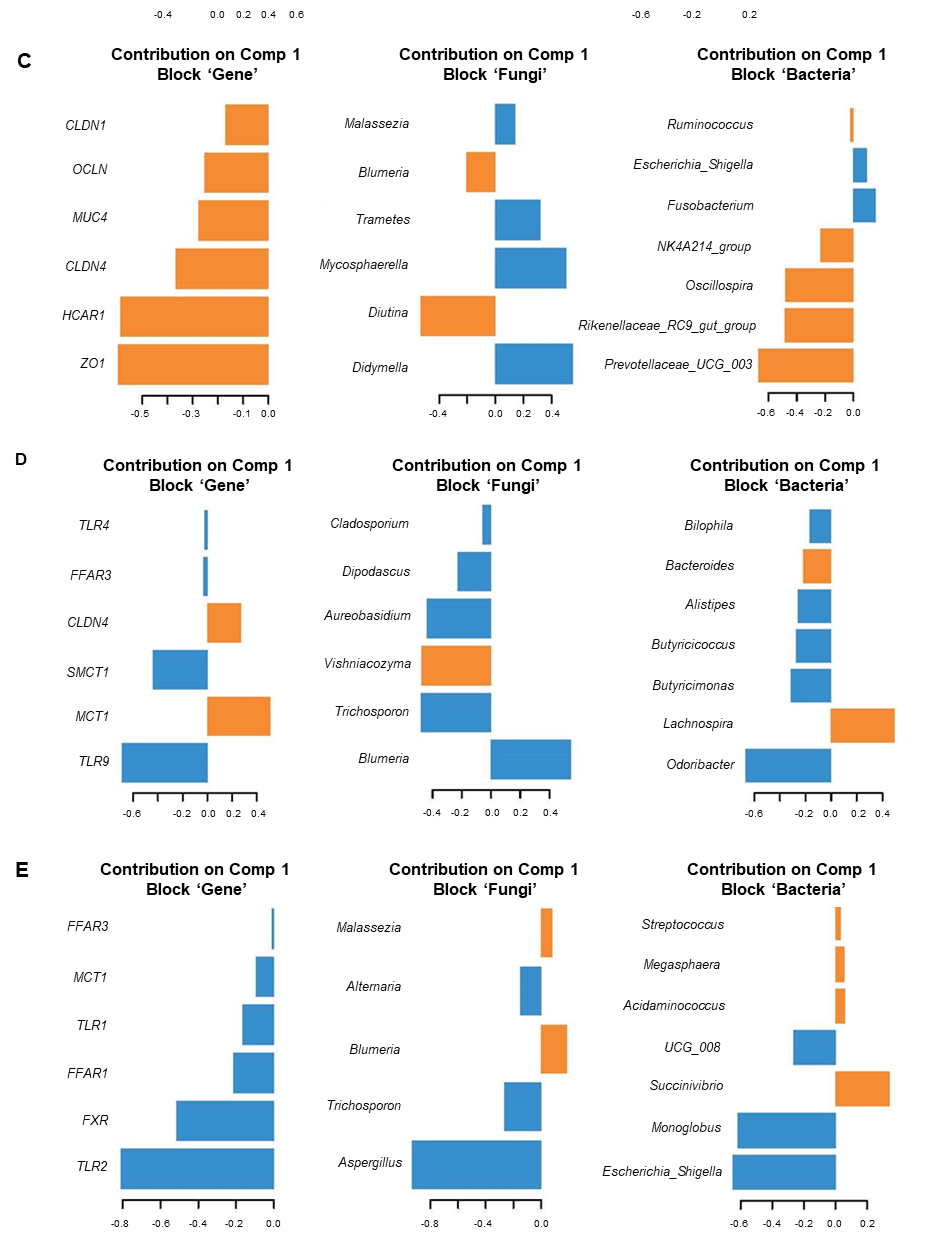


**Figure S3.** Circos plots of horizontal sparse partial least-squares-discriminant analysis displaying correlations in the cecum between the most discriminant bacterial and mycobial genera in the digesta and mucosal gene expression levels at day of life (**A**) 7 and (**C**) 21, and additionally fungal genera in the digesta at day of life (**B**) 14, (**D**) 28, (**E**) 31 and (**F**) 35. Positive and negative correlations (|*r*| > 0.4) are displayed by red and blue links, respectively. *FFAR1*, free fatty acid receptor 1; *FFAR3*, free fatty acid receptor 3; *FFAR4*, free fatty acid receptor 4; *HCAR1*, hydroxycarboxylic acid receptor 1; *MCT1*, monocarboxylate transporter 1; *SMCT1*, sodium coupled monocarboxylate transporter 1; *FXR*, farnesoid X receptor; *TLR1*, toll-like receptor 1; *TLR2*, toll-like receptor 2; *TLR4*, toll-like receptor 4; *TLR9*, toll-like receptor 9; *IAP*, intestinal alkaline phosphatase; *MUC4*, mucin 4; *CLDN1*, claudin 1; *CLDN4*, claudin 4; *OCLN*, occludin; *ZO1*, zonula occludens-1.

**
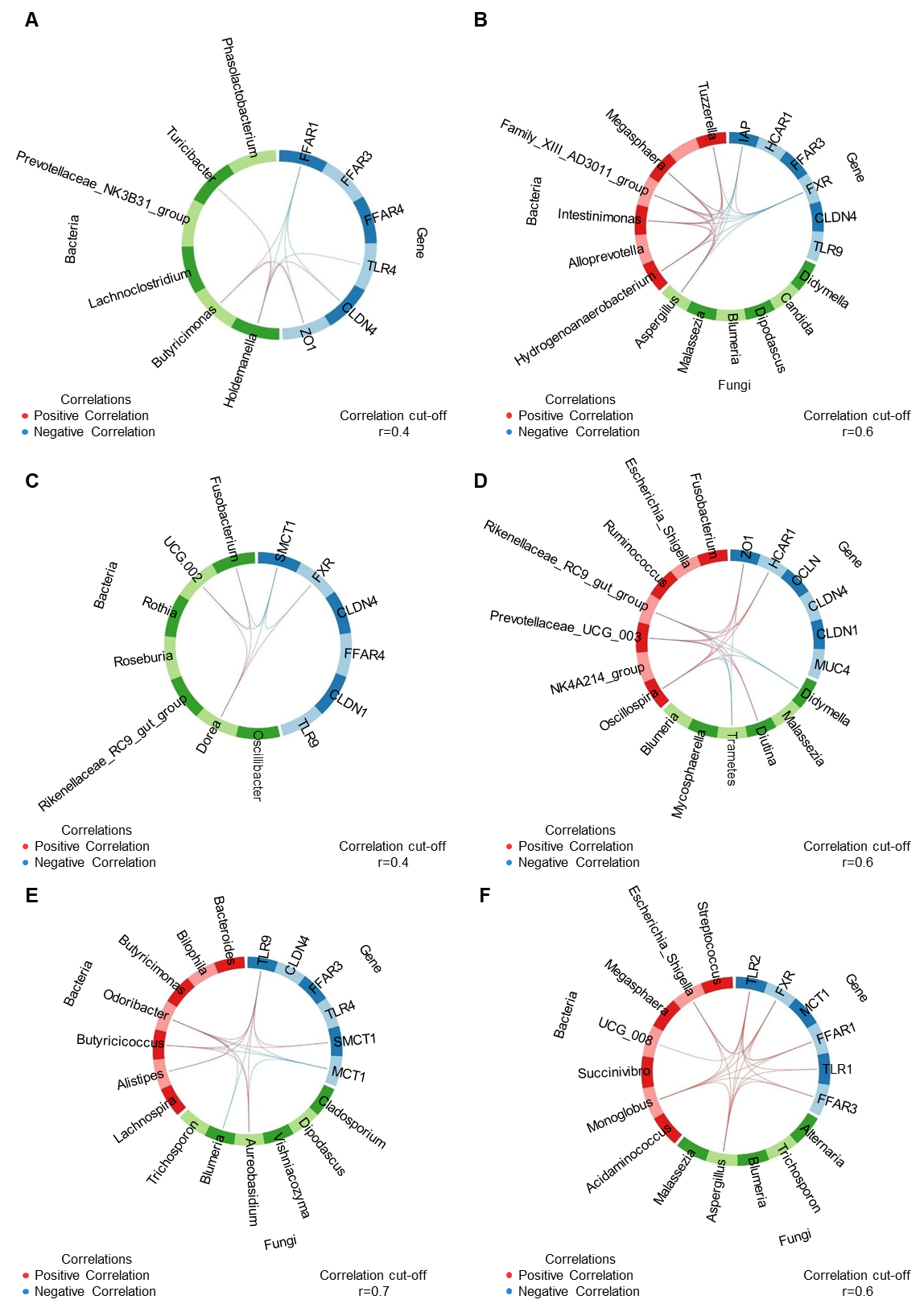
**

**References**

1. Muyzer, G., De Waal, E. C. & Uitterlinden, A. G. Profiling of complex microbial populations by denaturing gradient gel electrophoresis analysis of polymerase chain reaction-amplified genes coding for 16S rRNA. *Appl. Environ. Microbiol.* **59**, 695–700 (1993).

2. Sylvester, J. T., Karnati, S. K. R., Yu, Z., Morrison, M. & Firkins, J. L. Development of an assay to quantify rumen ciliate protozoal biomass in cows using real-time PCR. *J. Nutr.* **134**, 3378–3384 (2004).

3. Urubschurov, V., Büsing, K., Janczyk, P., Souffrant, W. B. & Zeyner, A. Development and evaluation of qPCR assay for quantitation of Kazachstania slooffiae and total yeasts occurring in the porcine gut. *Curr. Microbiol.* **71**, 373–381 (2015).

4. Zhou, M., Hernandez-Sanabria, E. & Le, L. G. Assessment of the microbial ecology of ruminal methanogens in cattle with different feed efficiencies. *Appl. Environ. Microbiol.* **75**, 6524–6533 (2009).

5. Klinsoda, J., Vötterl, J., Zebeli, Q. & Metzler-Zebeli, B. U. Alterations of the viable ileal microbiota of the gut mucosa-lymph node axis in pigs fed phytase and lactic acid-treated cereals. *Appl. Environ. Microbiol.* **86**, e02128-19 (2020).

6. Metzler-Zebeli, B. U., Ertl, R., Grüll, D., Molnar, T. & Zebeli, Q. Enzymatically modified starch up-regulates expression of incretins and sodium-coupled monocarboxylate transporter in jejunum of growing pigs. *Animal* **11**, 1180–1188 (2017).

7. Newman, M. A., Petri, R. M., Grüll, D., Zebeli, Q. & Metzler-Zebeli, B. U. Transglycosylated starch modulates the gut microbiome and expression of genes related to lipid synthesis in liver and adipose tissue of pigs. *Front. Microbiol.* **9**, 224 (2018).

8. Metzler-Zebeli, B. U. *et al.* Dietary calcium concentration and cereals differentially affect mineral balance and tight junction proteins expression in jejunum of weaned pigs. *Br. J. Nutr.* **113**, 1019–1031 (2015).

9. Metzler-Zebeli, B. U. *et al.* Short-chain fatty acids modulate permeability, motility and gene expression in the porcine fetal jejunum ex vivo. *Nutrients* **14**, 2524 (2022).

10. Arnaud, A. P. *et al.* Post-natal co-development of the microbiota and gut barrier function follows different paths in the small and large intestine in piglets. *FASEB J.* **34**, 1430–1446 (2020).
